# Phylogenomics supports monophyly of marsupial crustaceans: a journey to direct development

**DOI:** 10.1093/molbev/msag178

**Published:** 2026-08-13

**Authors:** Anna-Chiara Barta, Markus Grams, Heather Bracken-Grissom, Saskia Brix, Lívia M Cordeiro, Brittany Cummings, Stormie Collins, William J Farris, Sarah Gerken, Christoph G Höpel, Anne-Nina Lörz, Siena McKim, Kenneth Meland, Luise Kruckenhauser, Jørgen Olesen, Pedro A Peres, Stefan Richter, Regina Wetzer, Jason Williams, Kevin M Kocot, Martin Schwentner

**Affiliations:** Department of Evolutionary Biology, University of Vienna, Vienna 1030, Austria; Department of Zoology, Natural History Museum Vienna, Vienna 1010, Austria; Allgemeine & Spezielle Zoologie, Institut für Biowissenschaften, Universität Rostock, Rostock 18055, Germany; Institute of Environment, Department of Biological Sciences, Florida International University, Miami, FL 33181, USA; Senckenberg am Meer, German Center of Marine Biodiversity Research (DZMB), Hamburg 20146, Germany; Institute of Marine Ecosystem and Fishery Science (IMF), Center for Earth System Research and Sustainability (CEN), University of Hamburg, Hamburg 22767, Germany; Instituto Brasileiro de Estudos Subterrâneos, São Carlos, São Paulo, Brazil; Department of Biology, Florida Museum of Natural History, Gainesville, FL 32611, USA; Institute of Environment, Department of Biological Sciences, Florida International University, Miami, FL 33181, USA; Research and Collections, Natural History Museum of Los Angeles County, Los Angeles, CA 90007, USA; Department of Biological Sciences, University of Alaska Anchorage, Anchorage, AK 00508, USA; Allgemeine & Spezielle Zoologie, Institut für Biowissenschaften, Universität Rostock, Rostock 18055, Germany; Senckenberg am Meer, German Center of Marine Biodiversity Research (DZMB), Hamburg 20146, Germany; Institute of Marine Ecosystem and Fishery Science (IMF), Center for Earth System Research and Sustainability (CEN), University of Hamburg, Hamburg 22767, Germany; Department of Ecology, Evolution, and Marine Biology, University of California, Santa Barbara, CA 93106-9610, USA; Department of Biological Sciences, University of Bergen, Bergen 5020, Norway; Department of Evolutionary Biology, University of Vienna, Vienna 1030, Austria; Department of Zoology, Natural History Museum Vienna, Vienna 1010, Austria; Natural History Museum of Denmark, University of Copenhagen, Copenhagen 2100, Denmark; Institute of Environment, Department of Biological Sciences, Florida International University, Miami, FL 33181, USA; Allgemeine & Spezielle Zoologie, Institut für Biowissenschaften, Universität Rostock, Rostock 18055, Germany; Research and Collections, Natural History Museum of Los Angeles County, Los Angeles, CA 90007, USA; Department of Biology, Hofstra University, Hempstead, NY 11549-1140, USA; Department of Biological Sciences, University of Alabama, Tuscaloosa, AL 35487, USA; Department of Zoology, Natural History Museum Vienna, Vienna 1010, Austria

**Keywords:** molecular evolution, phylogenomics, peracarida, crustacea, malacostraca, molecular rate

## Abstract

Peracarida (marsupial crustaceans) represent one of the most diverse and ecologically important crustacean groups, yet their evolutionary relationships have long been debated. Here, we present the most comprehensive phylogenomic analysis of Peracarida to date, incorporating the relict taxa Thermosbaenacea, Mictacea, Ingolfiellida, and Spelaeogriphacea in a phylogenomic framework. Our results robustly confirm peracarid monophyly and recover a well-supported clade uniting Mancoida (Isopoda, Tanaidaca, Cumacea, Spelaeogriphacea) and Mictacea. We propose for Mictacea to be classified as part of the Mancoida, which is defined by shared developmental and morphological traits. Thermosbaenacea is suggested as sister group to all other Peracarida, although this position was not recovered in all analyses. The inferred phylogeny further suggests that evolution of Peracarida involved a transition from an intermediate “pseudodirect” developmental mode to the direct development seen in most lineages. We further show that the shift to extensive brood care within the marsupium, accompanied by the loss of a free-swimming larval stage, may have accelerated rates of molecular evolution across lineages. Together, these findings provide a robust evolutionary framework for this major malacostracan lineage and highlight how key reproductive innovations reshaped the genomic and life-history trajectories of the marsupial crustaceans.

## Introduction

**Figure 1:**
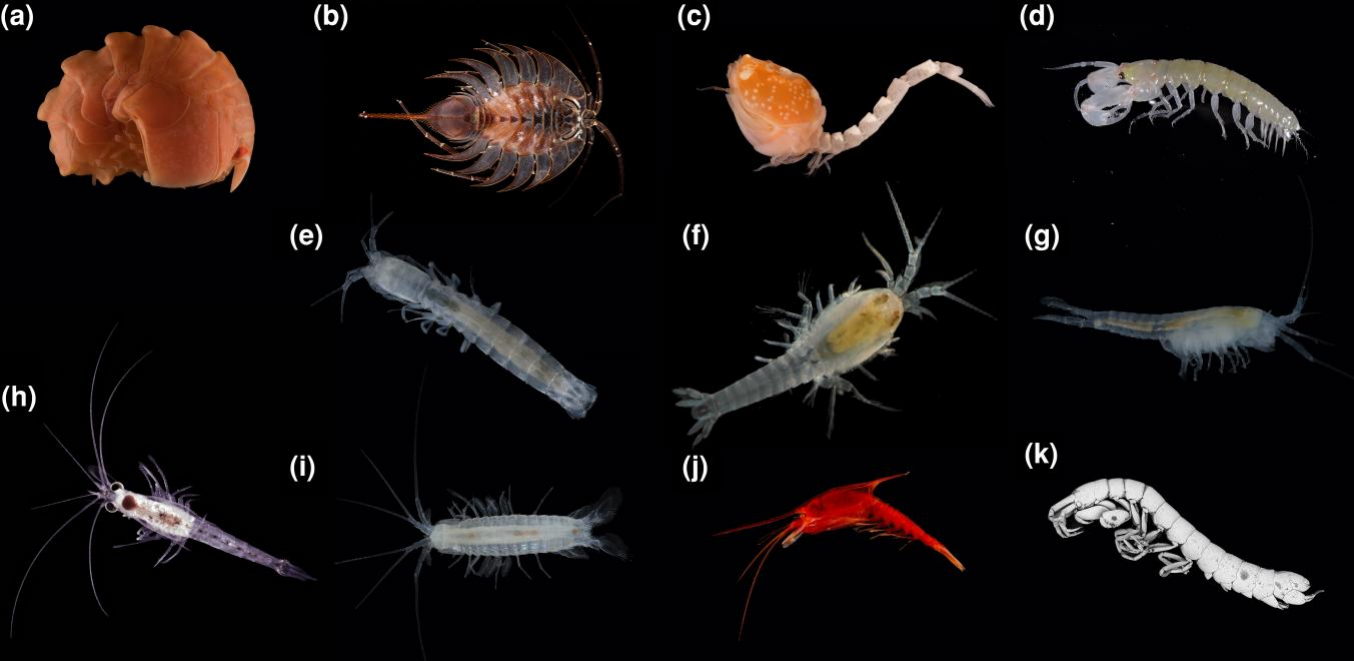
Morphological diversity of the studied orders within Peracarida. (a) *Epimeria inermis* (Amphipoda); (b) *Ceratoserolis meridionalis* (Isopoda); (c) *Cyclaspis gigas* (Cumacea); (d) Leptocheliidae (Tanaidacea); (e) *Stygiomysis clarkei* (Stygiomysida); (f) *Tulumella unidens* (Thermosbaenacea); (g) *Mictocaris halope* (Mictacea); (h) *Mysis relicta* (Mysida); (i) *Potiicoara brasiliensis* (Spelaeogriphacea); (j) *Gnathophausia zoea* (Lophogastrida); (k) *Ingolfiella* sp. (Ingolfiellida). Image credits: (a, b, and k) Kevin Kocot, (c) Victoria VanderBrandt, (d) Kamila Głuchowska, (e, f, g, and i) Jørgen Olesen, (h) Arild Hagen, (j) David Shale.

Peracarida (marsupial crustaceans) is one of the two major lineages of malacostracan crustaceans, rivaling Decapoda (shrimps, crabs, lobsters, and relatives) in both diversity and ecological scope. With ∼26,400 described species (WoRMS, accessed August 2025), they occupy nearly every marine, freshwater, and terrestrial habitat, where they are often abundant and have an important role in nutrient cycling. The group includes species-rich orders, such as Amphipoda, Isopoda, Cumacea, Tanaidacea, and Mysida, as well as several relict lineages with only a handful of species. These latter groups—Bochusacea (6 species), Ingolfiellida (54 species), Lophogastrida (53 species), Mictacea (1 species), Spelaeogriphacea (4 species), Stygiomysida (16 species), and Thermosbaenacea (45 species)—are largely confined to extreme or isolated environments, such as caves, subterranean aquifers, and the deep sea. Despite their ecological importance and diversity, fundamental questions about the group's evolutionary history remain unanswered (Fig. 1).

Traditional morphology-based phylogenetic investigations generally support peracarid monophyly, based on shared derived characters, such as the brood pouch (marsupium; Richter and Scholtz 2001; Poore 2005; Wills et al. 2009; Wirkner and Richter 2010; Grams et al. 2025). However, molecular studies have yielded conflicting results regarding peracarid monophyly. Single-gene analyses based on 18S or 28S rDNA (eg Jarman et al. 2000; Spears et al. 2005; Meland and Willassen 2007; Jenner et al. 2009) consistently excluded Mysida from Peracarida. These studies recovered mysids in a clade with Euphausiacea, Stomatopoda, and/or Syncarida. In contrast, phylogenomic studies (eg Schwentner et al. 2018; Höpel et al. 2022; Bernot et al. 2023; Yu et al. 2024; Dreyer et al. 2025) have consistently recovered a monophyletic Peracarida. However, most of these studies focused on broader pancrustacean relationships and had substantial gaps in peracarid taxon sampling; in particular, the above-mentioned relict lineages, except for Lophogastrida and/or Stygiomysida, were missing. Two recent studies using ultraconserved elements focusing on Peracarida have been published. While one is mainly a proof-of-concept study (Cannizzaro and Berg 2026), the other has a relatively dense peracarid sampling (Iwasa-Arai et al. 2026).

Central to this debate is the unique reproductive strategy of Peracarida that involves their ventral marsupium, where embryos develop directly into juvenile forms without free-living larval stages. Exceptions occur in two groups of parasitic isopods (Epicaridea and Gnathiidae) that have dispersive larval stages (eg Williams and Boyko 2012; Baeza 2015; Schädel et al. 2019; Williams et al. 2022). Peracarid direct development contrasts with that of other malacostracan crustaceans, such as decapods, which typically have complex dispersive larval phases (Martin et al. 2014). While most peracarid groups release juveniles that look like miniature adults (“manca” stages or similar), Mysidacea (Mysida, Stygiomysida, and Lophogastrida) produce an intermediate nauploid (characterized by an advanced development of the first head appendages, ie antennula, and antenna) that completes development within the pouch (pseudodirect development; San Vincente et al. 2014; Jirikowski et al. 2015). The monophyly of Mysidacea remains one of the most contentious questions within Peracarida. While morphological studies support its monophyly (Richter and Scholtz 2001; Poore 2005; Wirkner and Richter 2010; Grams et al. 2025), molecular analyses based on 18S or 28S rDNA reject this hypothesis, most notably by recovering Mysida outside of Peracarida and phylogenetically distant to Lophogastrida and Stygiomysida (Jarman et al. 2000; Spears et al. 2005; Meland and Willassen 2007).

Over the years, various morphological studies have supported a sister group relationship of the two most diverse taxa, Amphipoda and Isopoda, based on shared (but mostly reductive) characteristics, such as sessile eyes, reduced carapace, and absence of thoracic exopods (eg Wagner 1994; Wills 1998; Poore 2005). However, this view has been challenged by both morphological and molecular studies that instead recover a clade uniting Isopoda with Cumacea, Tanaidacea, and Spelaeogriphacea (eg Siewing 1956; Richter and Scholtz 2001; Schwentner et al. 2018; Bernot et al. 2023; Iwasa-Arai et al. 2026) named Mancoida for their shared “manca” larval stage (Watling 1981). A manca-like larva (ie lacking posterior thoracopods) is also known from some other peracarid taxa: Mictacea and Bochusacea (both discovered only after Watling 1981, defined the taxon Mancoida), but also Thermosbaenacea. In Thermosbaenacea, hatchlings lack the last one to two pairs of thoracopods (Stella 1951; Zilch 1972; Wagner 1994; Olesen et al. 2015), and in two species (*Thermosbaena mirabilis* and *Salishbaena kootenai*), this lack of the last thoracopods is retained in adults. Thermosbaenaceans also show a striking exception to the ventral marsupium by brooding their young in a dorsal carapace chamber (Olesen et al. 2015). Based on this fundamental difference, it has been proposed that Thermosbaenacea is not part of Peracarida (eg Siewing 1956, 1958, 1963; Richter and Scholtz 2001). However, most contemporary studies place Thermosbaenacea as nested within Peracarida and closely related to the mancoid taxa (eg Wagner 1994; Wills 1998; Poore 2005; Spears et al. 2005; Meland and Willassen 2007; Wilson 2009; Wirkner and Richter 2010; Ashford et al. 2020). The only phylogenomic study to date that included Thermosbaenacea placed it as sister group to all other Peracarida (Iwasa-Arai et al. 2026), a position that would be in line with previous morphology-based hypotheses (eg Siewing 1956, 1958, 1963; Richter and Scholtz 2001).

Testing peracarid monophyly, clarifying the relationships of Mysidacea, evaluating hypotheses such as Mancoida, and solving the phylogenetic position of Thermosbaenacea are key prerequisites for understanding the evolutionary transformations leading to brood care within a marsupium and the evolution of direct, nondispersive development. To resolve these crucial questions of peracarid evolution, we employed transcriptomic and genomic data in a comprehensive phylogenomic approach designed to mitigate systematic error from heterogeneous rates of molecular evolution and compositional bias. We generated a densely sampled dataset of 156 malacostracans, with a core focus on 128 peracarids, including all orders except the rarely sampled, deep-sea taxon Bochusacea. This includes a comprehensive representation of Mysidacea, as well as the first phylogenomic data for the relict taxa Spelaeogriphacea and Ingolfiellida. In addition, our expansive sampling within Amphipoda, Cumacea, Isopoda, Mysida, and Tanaidacea provides a robust higher-level phylogenetic framework for each of these species-rich taxa. Data were analyzed under multiple analytical frameworks and evolutionary models to assess the robustness of the phylogenetic inference.

## Results

Our phylogenomic workflow incorporated genomic and transcriptomic data from 156 taxa, comprising 92 publicly available datasets (NCBI SRA; transcriptomes and genomes) and 64 newly sequenced transcriptomes. These include 128 peracarid and 28 other malacostracan taxa, providing a broad representation across all nonperacarid orders to test peracarid monophyly and identify the potential sister group to Peracarida. From these data, we constructed four complementary phylogenomic matrices: (i) a dataset based on Arthropoda BUSCO genes [673 loci; concatenated alignment length = 271,685 amino acids {AA}; 24% missing data {= gaps out of total alignment positions}; mean locus length = 404 AA; range = 101 to 66,096 AA; 240,425 parsimony-informative sites], (ii) a dataset with de novo orthogroup identification (ORTHO dataset: 511 loci; 189,938 AA; 29.7% missing data; mean locus length = 372 AA; range = 101 to 6,096 AA; 177,520 parsimony-informative sites) and their respective signal-optimized subsets, (iii) BUSCO-50% (337 loci; 172,103 AA; 25.6% missing data; mean locus length = 511 AA; range = 120 to 6,096 AA; 154,225 parsimony-informative sites), and (iv) ORTHO-50% (255 loci; 95,481 AA; 29% missing data; mean locus length = 374 AA; range = 108 to 1,175 AA; 86,209 parsimony-informative sites), which were created by retaining the 50% most phylogenetically informative loci from each parent dataset. Phylogenetic reconstruction employed maximum likelihood (ML), Bayesian inference (BI), maximum parsimony (MP), and coalescent methods for a total of six approaches: ML in IQ-TREE 2 with (i) ModelFinder used to find the best-fitting model for each gene; (ii) GHOST edge-unlinked mixture model, which accounts for heterotachous evolution; (iii) PMSF site-specific frequency model (LG + C20 + F + G model); (iv) ASTRAL-III coalescence; (v) RAxML analysis (PROTOGAMMAAUTO model) of Dayhoff-recoded matrices; and (vi) MP in TNT. BI (PhyloBayes MPI) was conducted on the BUSCO-50% and ORTHO-50% datasets with a reduced taxon set (*n* = 62 taxa) with extended MCMC chains (>10,000 generations). We rooted all trees with Leptostraca, which is the sister group to all remaining Malacostraca (Richter and Scholtz 2001; Schwentner et al. 2018; Bernot et al. 2023; Dreyer et al. 2025).

**Figure 2:**
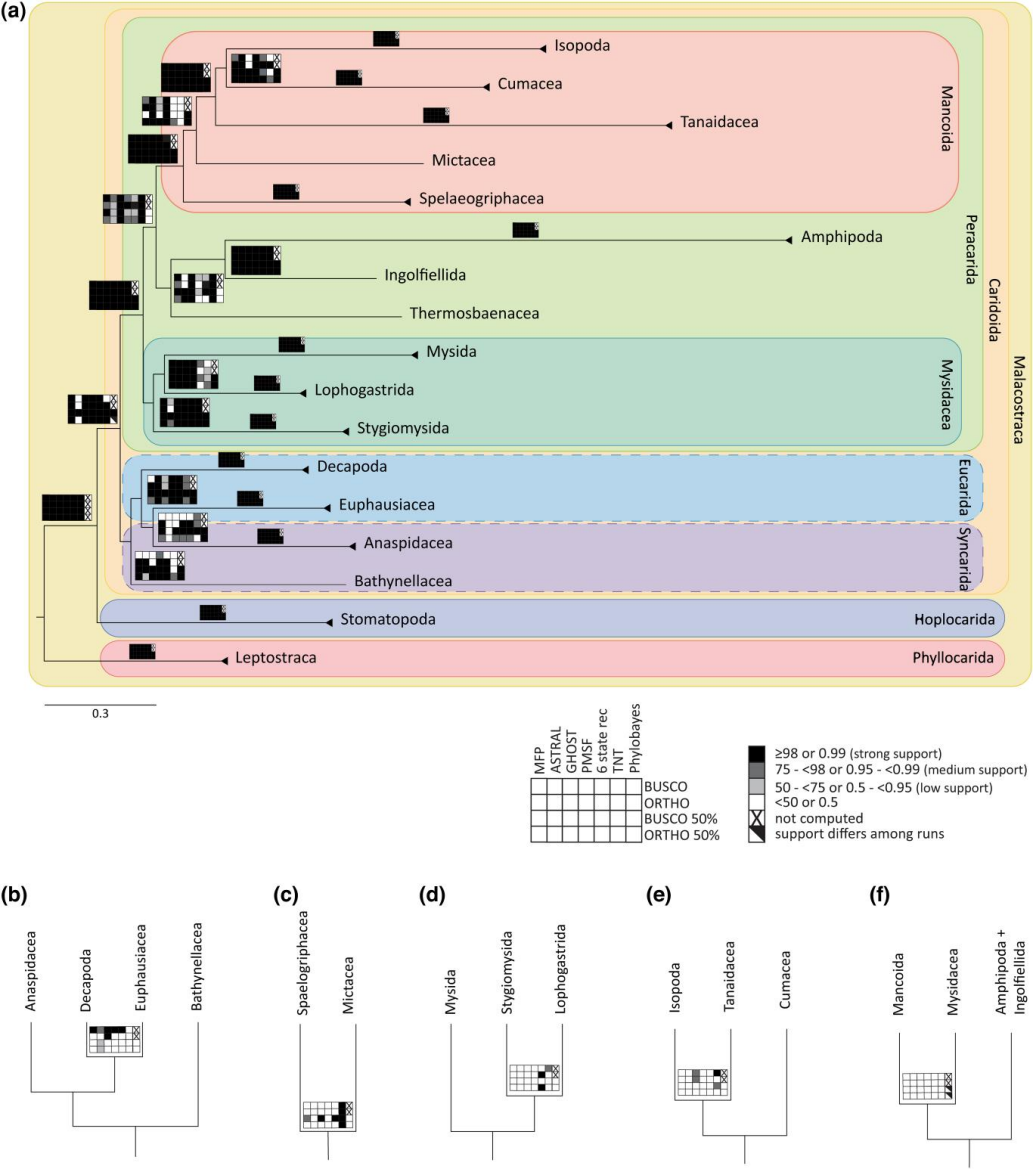
Summary of phylogenetic analyses. (a) Topology based on ORTHO-50% matrix comprising 255 phylogenetically informative loci and 95,481 AA positions analyzed with IQ-TREE MFP. Support values for all analyses are plotted on or below respective branches as specified in the legend at the lower-right corner. Nodes are collapsed to show only the respective orders (matrix of support values are shown on each respective branch). Detailed relationships within orders are depicted in Figs. S2 to S8, and all original species trees are depicted in Figs. S9 to S34; (b to f) relevant alternative topologies.

**Figure 3:**
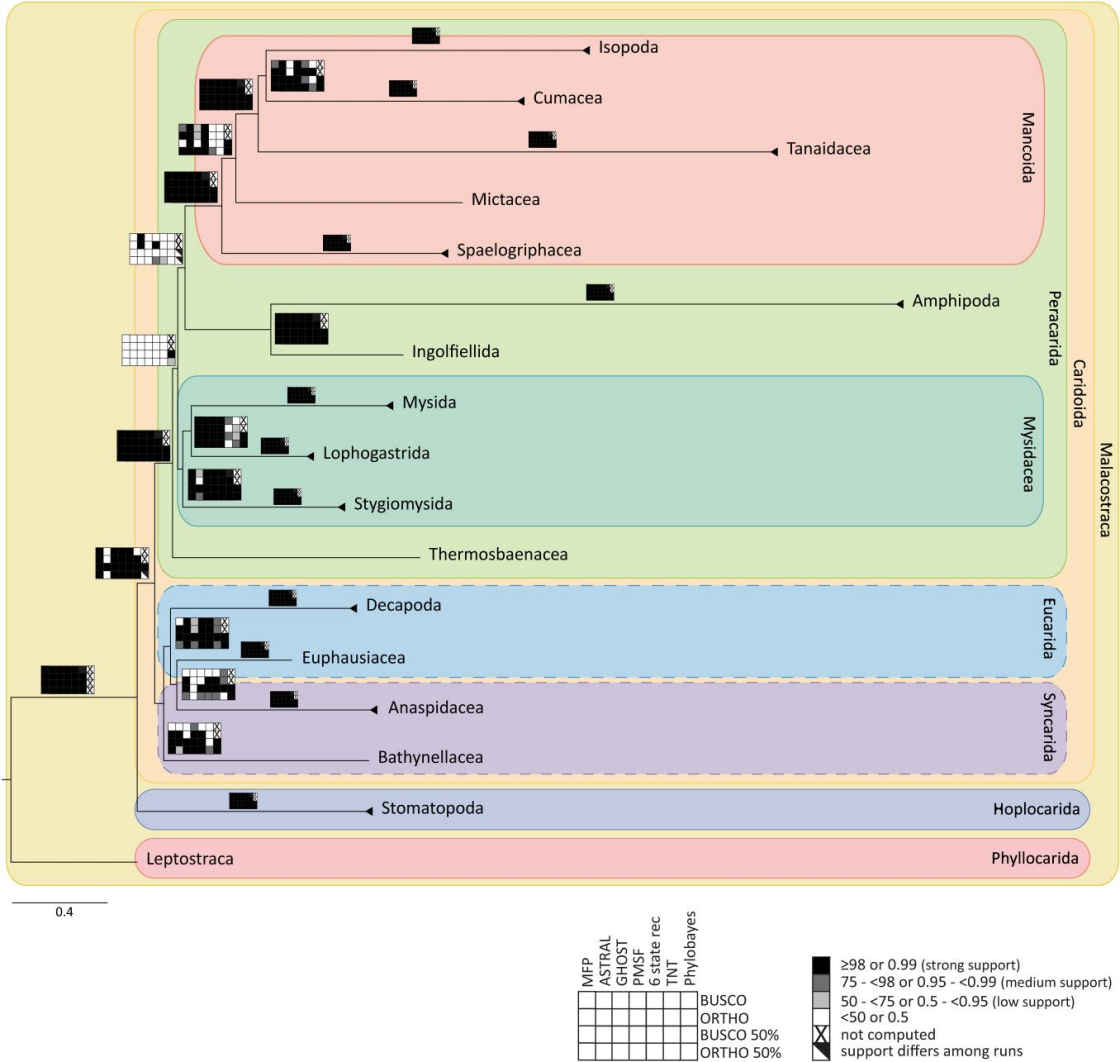
Summary of phylogenetic analyses. Topology based on a reduced taxon set (*n* = 62) of ORTHO-50% matrix comprising 251 phylogenetically informative loci and 93,319 AA positions analyzed with PhyloBayes GAT-GTR. Support values for all analyses are plotted on or below respective branches as specified in the legend at the lower-right corner. Nodes are collapsed to show only the respective orders (matrix of support values are shown on each respective branch). Detailed relationships within orders are depicted in Figs. S2 to S8, and all original species trees are depicted in Figs. S9 to S34.

**Figure 4:**
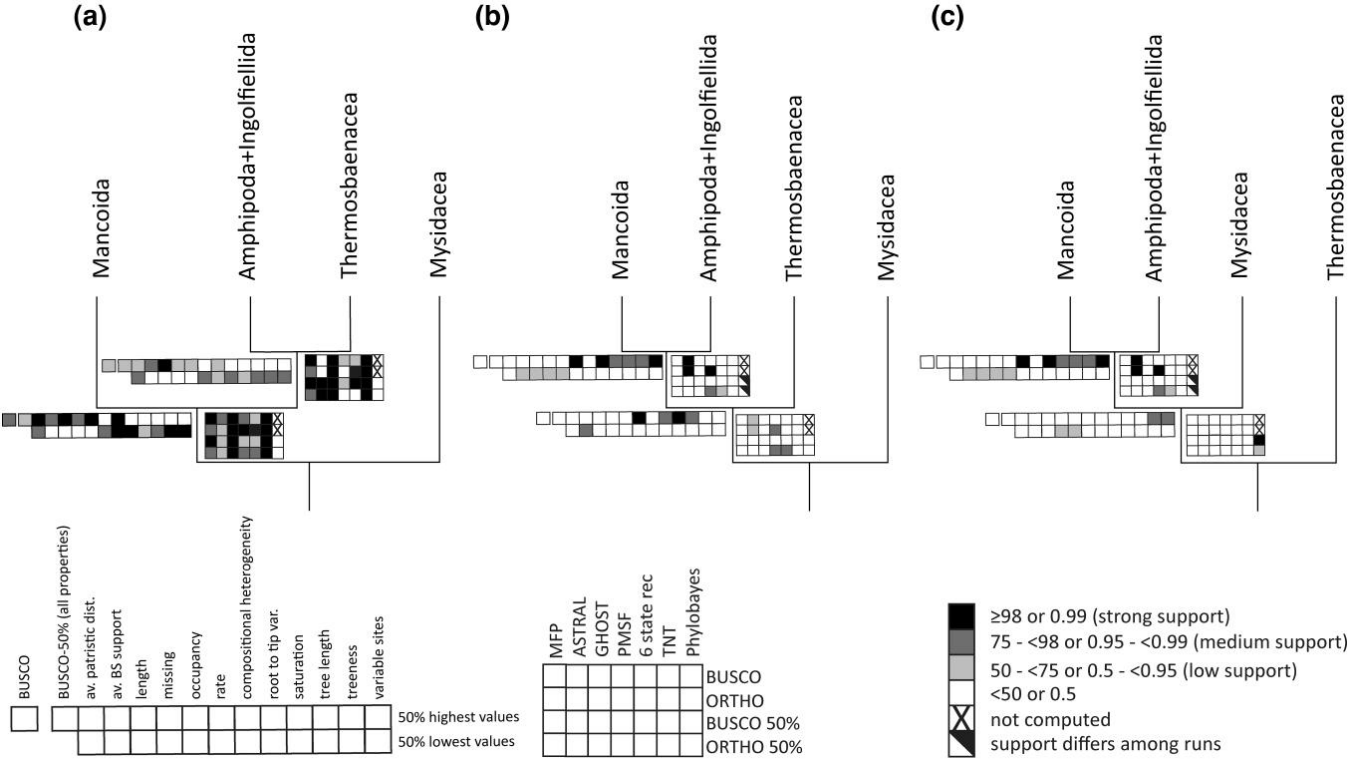
Alternative topologies for the position of Thermosbaenacea recovered when subsampling for different locus properties. Support values for subsampled datasets are shown below and to the left of each branch; support values for the original full-dataset analyses are shown below and to the right.

**Figure 5:**
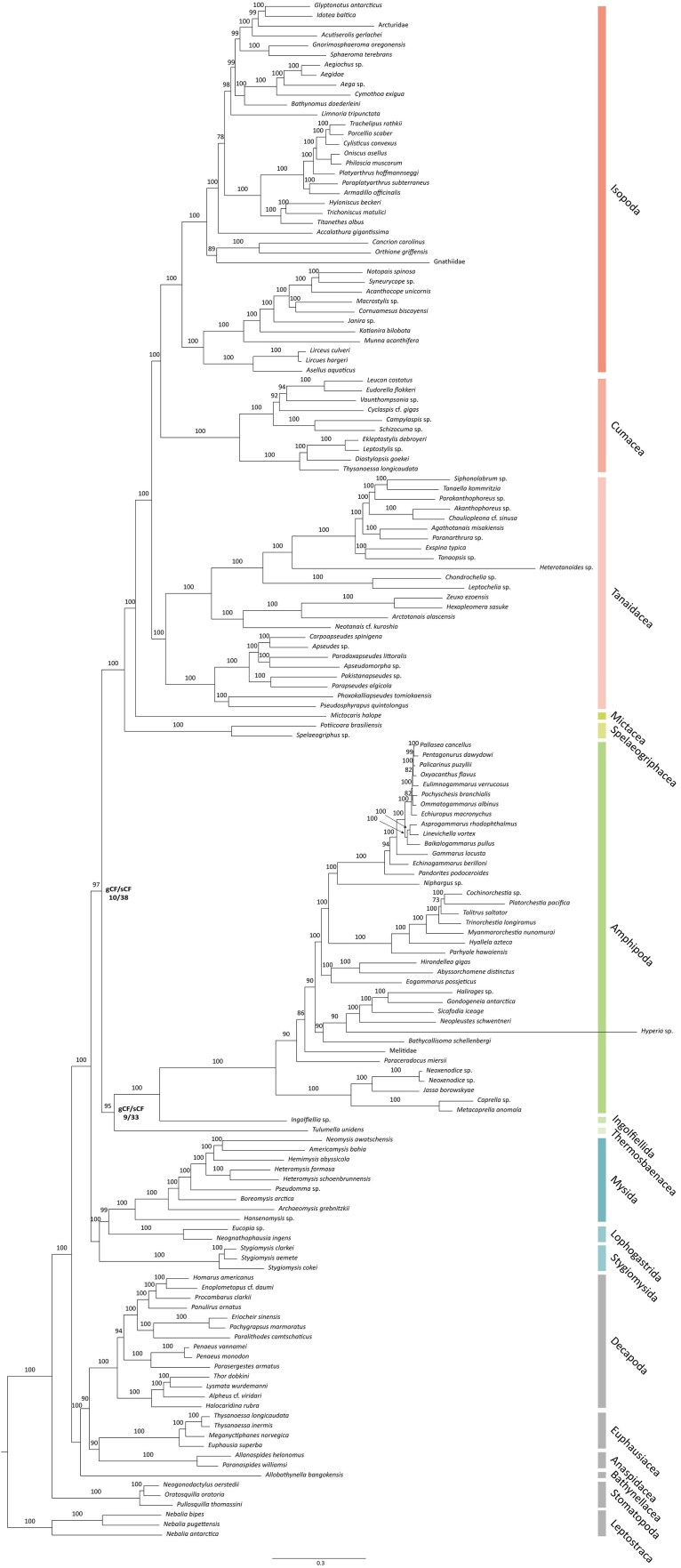
Phylogenetic analysis with ORTHO-50% matrix comprising 255 phylogenetically informative loci and 95,481 AA positions analyzed with IQ-TREE MFP. Support values for all analyses are plotted on or below respective branches. gCF/sCF supporting the position of *T. unidens* (Thermosbaenacea) are depicted on the corresponding nodes.

**Figure 6:**
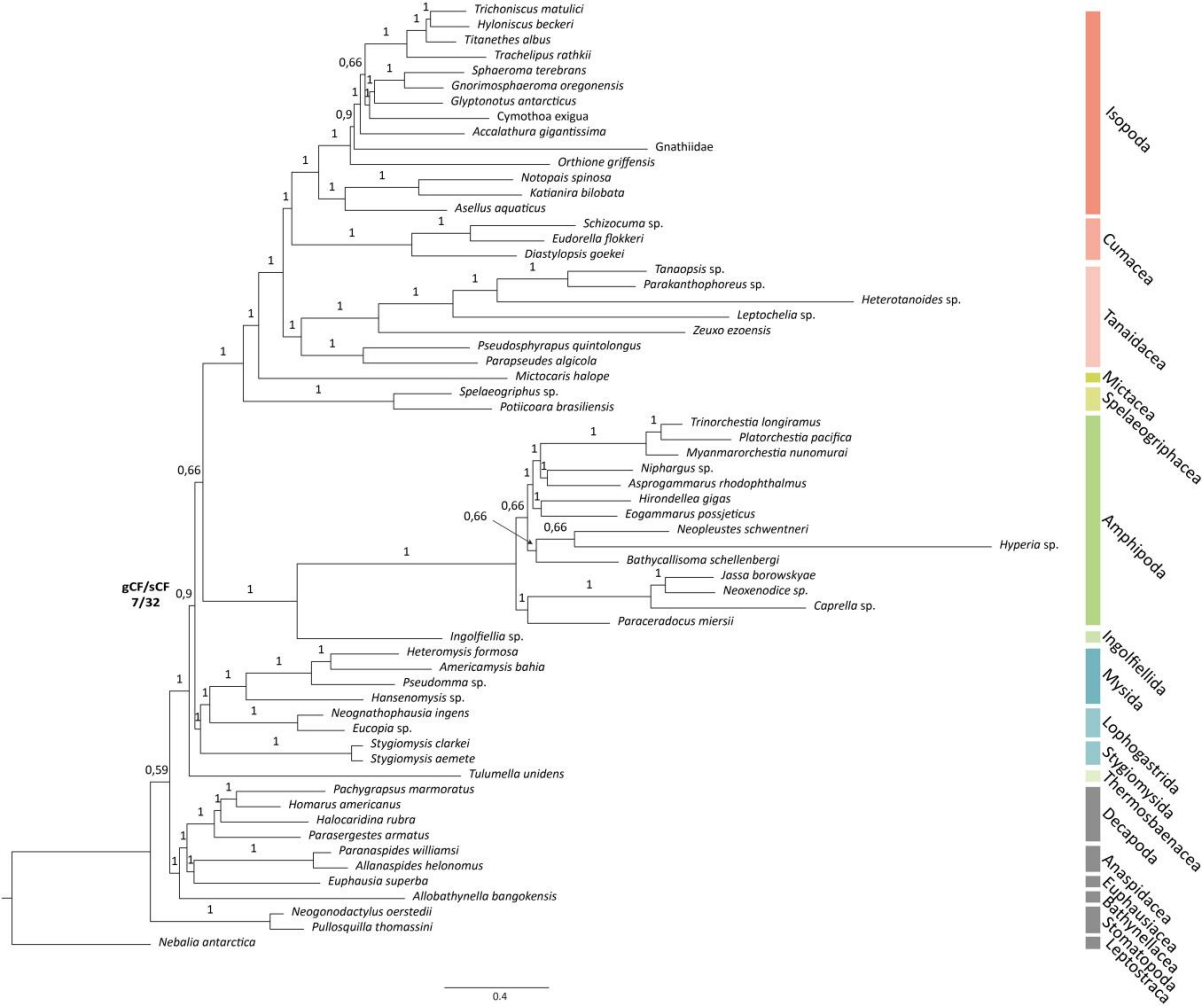
Phylogenetic analysis of ORTHO-50% matrix on a reduced taxon set (*n* = 62) comprising 251 phylogenetically informative loci and 93,319 AA positions analyzed with PhyloBayes GAT-GTR. Support values for all analyses are plotted on or below respective branches. gCF/sCF supporting the position of *T. unidens* (Thermosbaenacea) are depicted on the corresponding nodes.

All datasets and analytical methods congruently supported the monophyly of Peracarida and all peracarid orders with strong support (Figs. 2 and 3; detailed relationships within orders are presented in Figs. S2 to S8, and all original species trees are presented in Figs. S9 to S34). Within Peracarida, Mysidacea (Mysida + Lophogastrida + Stygiomysida) formed a monophyletic group with strong support (≥98 or 0.99); only some ASTRAL analyses had lower support for Mysidacea. Notably, the ASTRAL analysis of the ORTHO dataset failed to resolve deep peracarid relationships, resulting in a polytomy at the ordinal level. The majority of analyses recovered Mysidacea as the sister group to all other Peracarida; however, support for this placement varied. This was largely due to the unstable position of Thermosbaenacea, which was either recovered as the taxon sister to (i) Amphipoda + Ingolfiellida (the most commonly retrieved position), (ii) all non-Mysidacea Peracarida (recovered in some ASTRAL, PMSF, and six-state recoding analyses), or (iii) all other Peracarida (only in the PhyloBayes CAT-GTR analysis) (Figs. 2 and 3). The unstable position of Thermosbaenacea is further underlined by the exceptionally low values for gene concordance factor (gCF) and site concordance factor (sCF) for all positions of Thermosbaenacea (Figs. 5 and 6). To investigate the impact of different potential sources of systematic error on the position of Thermosbaenacea, we subsampled the BUSCO dataset for 12 different gene properties and analyzed each of these subsets with the PMSF model. For each gene property, two matrices were generated and analyzed, resulting in a total of 24 matrices: the loci with the 50% highest and the 50% lowest values for each property. The PMSF analysis of the complete BUSCO dataset recovered topology A (Thermosbaenacea as sister to Amphipoda + Ingolfiellida), and also for each of the tested gene properties, one subset (either the 50% highest or lowest) recovered that topology (Fig. 4). However, for 10 of the 12 properties, the respective other subset recovered either topology B (sister to all nonmysidacea-Peracarida; lower average bootstrap support, higher rate of evolution, higher root to tip variation, higher saturation, and higher tree length) or C (sister to all other Peracarida; lower proportion of missing data, lower occupancy, higher treeness, and higher proportion of variable sites) (Fig. 4).

Amphipoda and Ingolfiellida were recovered as sister groups in all analyses, with strong to maximal support. Similar strong support was recovered for a clade uniting Mancoida (Isopoda + Cumacea + Tanaidacea + Spelaeogriphacea) with Mictacea. The predominant topology placed Mictacea as the sister group to a strongly supported Isopoda + Cumacea + Tanaidacea clade, although some analyses recovered a clade of Mictacea + Spelaeogriphacea as sister to the remaining Mancoida (the latter mainly recovered in six-state recoding and TNT analyses). In most analyses, Isopoda and Cumacea were recovered as sister groups with strong support, except for some ASTRAL and TNT analyses, where Isopoda formed a clade with Tanaidacea, albeit with varying support. In general, discrepancies in relationships among orders were mainly observed among different phylogenetic inference methods and/or models and less among the matrices inferred by very different orthology-inference strategies.

Within Isopoda, our results robustly place Asellota as the sister group to all other isopods (Figs. S5 and S6). The subsequent divergence involves Gnathiidae (Cymothoida) and Epicaridea, which form a clade in some analyses (Fig. S5) but emerge as separate branches in others (Fig. S6), consistently resulting in a paraphyletic Cymothoida. Oniscidea was mostly recovered as the sister to all remaining Isopoda, followed by *Accalathura gigantissima* (Cymothoida); however, in some analyses, including all ORTHO-50% analyses, *A. gigantissima* diverges before Oniscidea. Relationships among the remaining sampled isopods are ambiguous. In the most commonly obtained topology, Limnoriidea branches off first, followed by the remaining Cymothoida (*Aegiochus* sp., Aegidae, *Cymothoa exigua*, *Bathynomus doederleini*), and paraphyletic Sphaeromatidea (*Acutiserolis gerlachei*, *Gnorimosphaeroma oregonensis*, *Sphaeroma terebrans*) with respect to Valvifera (Fig. S5). Notably, the serolid *A. gerlachei* (Sphaeromatidea) consistently groups with Valvifera with strong support in all analyses.

Within Cumacea, Diastylidae was consistently recovered as the sister taxon to all other sampled cumaceans, with Nannastacidae as the sister taxon of Bodotriidae + Leuconidae with strong support (Fig. S4). Notably, Bodotriidae was consistently resolved as paraphyletic with respect to Leuconidae, usually with strong support.

All tanaidacean superfamilies were recovered asmonophyletic with strong support (Fig. S8). Apseudomorpha was robustly placed as the sister group to the suborder Tanaidomorpha. Within Tanaidomorpha, the superfamily Neotanaoidea (represented by *Neotanais* cf. *kuroshio*) was consistently recovered as the sister taxon to Tanaidoidea.

Within Amphipoda, some relationships were consistently recovered; however, the backbone within the order remained poorly resolved, and the position of several key taxa varied across analyses (Figs. S2 and S3). This may be largely due to the instability of Hyperiidea (represented by *Hyperia* sp.), a long branched taxon that was recovered as either the sister taxon to all other sampled Amphipoda (Fig. S3) or within a clade containing calliopioid, iphimedioid, and amphilochioid taxa (Fig. S2). The established subordinal classification was not supported with Senticaudata and Amphilochidea paraphyletic in all analyses. Talitroidea was monophyletic and consistently formed a clade with representatives of Hyloidea (*Hyalella azteca* and *Parhyale hawaiensis*), rendering the latter paraphyletic. The two Hadzioidea species formed a monophyletic group in the majority of analyses (Fig. S3). A clade consisting of a monophyletic Calliopioidea, Iphimedioidea (*Sicafodia iceage*), and Amphilochoidea (*Neopleustes boecki*) was consistently recovered. The positions of lysianassoid taxa were highly variable. *Bathycallisoma schellenbergi* (Lysianassoidea) was often associated with the calliopioid–iphimedioid–amphilochoid clade (Fig. S2) and was recovered with other lysianassoids in only a single analysis (Fig. S3). While the infraorder Corophiida was monophyletic, the photoid *Jassa borowskyae* clustered with Caprelloidea, rendering the latter paraphyletic. Gammaroidea formed a well-supported clade in all analyses (only *Eogammarus possjeticus* consistently grouped outside), with *Niphargus* sp. (Crangonyctoidea) as its sister group.

Within Mysidacea, Lophogastrida was recovered as the sister group to Mysida with strong support, except in the TNT analyses (Fig. S7), where support was moderate to low. Analyses of the Dayhoff-recoded ORTHO and ORTHO-50% matrices, however, suggest a closer relationship between Lophogastrida and Stygiomysida, each with strong support (only the ASTRAL ORTHO analysis resulted in a polytomy of Stygiomysida, Mysida + Lophogastrida, and the other Peracarida). Relationships within Mysida were stable and highly supported across all analyses (Fig. S7). *Hansenomysis* sp. (Petalophthalmidae) was consistently recovered as the sister group to all other sampled Mysida (representing the family Mysidae).

Stomatopoda emerged as the sister group to Caridoida (Syncarida + Eucarida + Peracarida) in most analyses (Figs. 2 and 3), with high support (BS ≥ 98; PP ≥ 0.99), only some ASTRAL analyses and one chain of the PhyloBayes ORTHO-50% analysis clustered Stomatopoda together with Decapoda + Euphausiacea + Anaspidacea + Bathynellacea. TNT analysis of the BUSCO dataset recovered Stomatopoda as the sister group to Decapoda + Euphausiacea + Anaspidacea and Bathynellacea as the sister group to Caridoida (Fig. S14).

**Figure 7:**
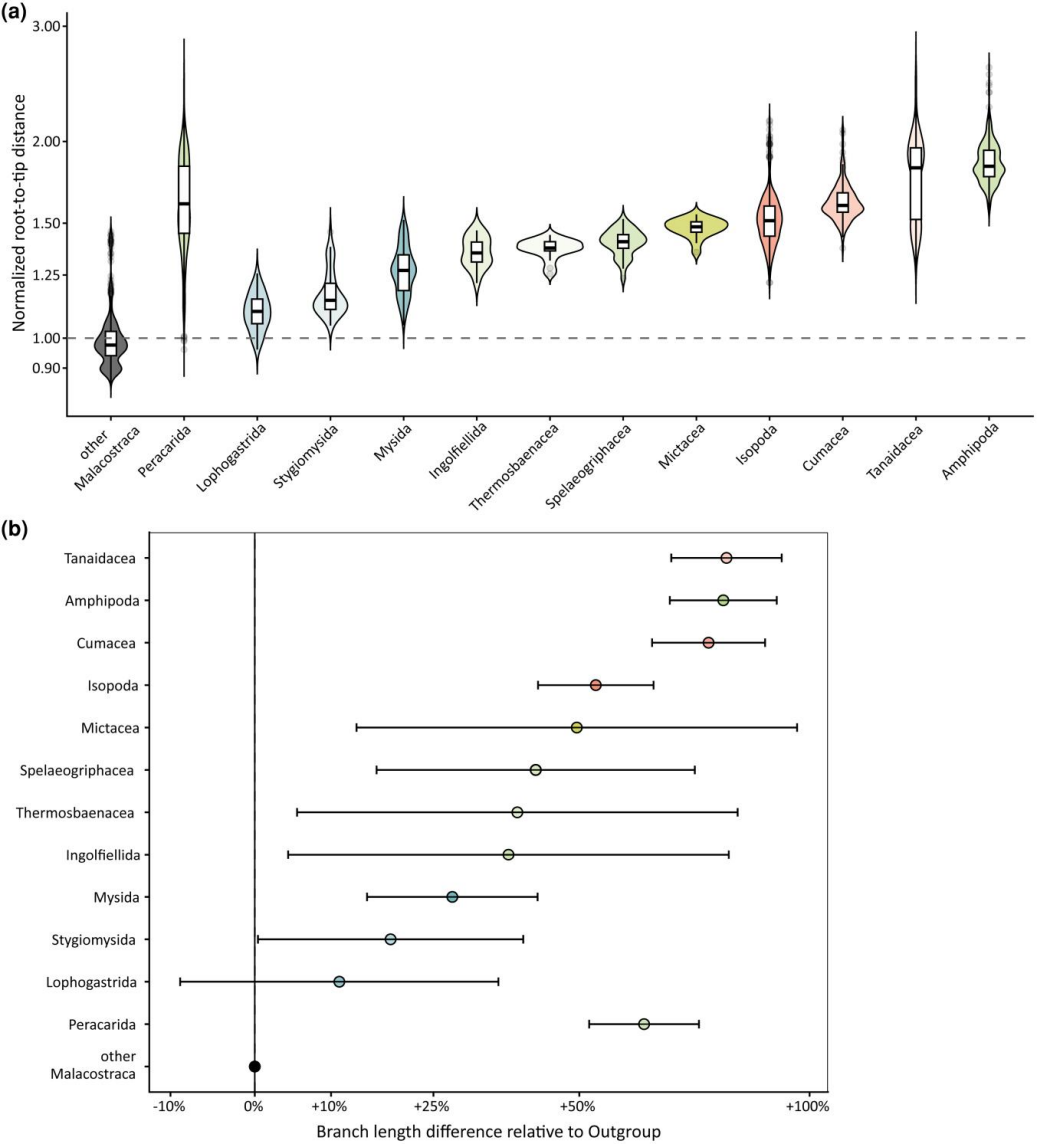
Summary of evolutionary rate analyses. (a) Comparison of RTT distances across taxonomic groups. RTT values were scaled within each phylogenetic tree, except ASTRAL and TNT analyses, by dividing species-specific RTT by the mean outgroup RTT from the same tree. Violin plots show the full distribution of normalized RTT values, while embedded boxplots indicate medians and interquartile ranges. (b) Forest plot of RTT distance differences between Peracarida orders and the other Malacostraca (Decapoda, Bathynellacea, Anaspidacea, and Euphausiacea). Points show estimated marginal mean differences (log-transformed RTT) from the linear mixed model, with 95% CIs. Values above zero indicate longer RTT relative to the outgroup; intervals not overlapping zero denote significant differences (Tukey-adjusted, *P* < 0.05). ASTRAL and TNT phylogenetic inferences were not included in this plot.

Evolutionary rate comparisons revealed that peracarids exhibit root-to-tip (RTT) values that are 63% longer on average than for all other Malacostraca (Fig. 7, LMM model 1: estimate = 0.48 ± 0.04, *df* = 147.01, *t* = 14.01, *P* < 2 × 10^−16^, Table S37), when excluding ASTRAL analyses. This trend is consistent across all 18 phylogenetic analyses (Fig. S35). When examining RTT at the order level, we detected variation among Peracarida orders (Fig. 7, LMM model 2 in Table S37). Focusing on analyses excluding ASTRAL, most orders exhibited significantly longer RTT compared to nonperacarid malacostracans, including Amphipoda (80% longer), Cumacea (76% longer), Ingolfiellida (27% longer), Isopoda (53% longer), Mysida (28% longer), Spelaeogriphacea (42% longer), Mictacea (50% longer), Stygiomysida (18% longer), and Tanaidacea (80% longer) (*P* < 0.05 in LMM model 2; see Table S37 for details). In contrast, only Lophogastrida (11% longer) showed RTT values not significantly different from the outgroup (*P* > 0.05), while other previously nonsignificant orders reached significance once ASTRAL trees were excluded. Random effects indicate that RTT scaling varies more strongly across trees (variance_Tree_ = 0.945) than species (variance_species_ = 0.007), representing that differences among species remain consistent even when different methods have been used for phylogenetic inference. Notably, ASTRAL trees represent the set showing consistently shorter RTT than other phylogenetic reconstructions (Fig. S35), and the overall evolutionary rate was only 56% longer when ASTRAL trees were included (Fig. S36, LMM model 1: estimate = 0.44 ± 0.03, *df* = 147.12, *t* = 12.11, *P* < 2 × 10^−16^). Results concerning Lophogastrida, Ingolfiellida, Mictacea, Spelaeogriphacea, Stygiomysida, and Thermosbaenacea should be interpreted with caution, as these groups are represented by three or fewer species in our dataset (Fig. 7).

## Discussion

This study, covering 128 peracarid and 28 nonperacarid taxa and 255 to 673 loci per matrix, robustly recovered Peracarida as a monophyletic group, resolving a key controversy surrounding one of the most diverse and ecologically significant crustacean lineages. This result is consistent with previous morphological studies (eg Richter and Scholtz 2001; Poore 2005; Wirkner and Richter 2010; Grams et al. 2025), as well as phylogenomic analyses (eg Schwentner et al. 2018; Lozano-Fernandez et al. 2019; Höpel et al. 2022; Bernot et al. 2023; Dreyer et al. 2025; Iwasa-Arai et al. 2026). However, earlier phylogenomic analyses lacked several rare relict taxa, Thermosbaenacea, Mictacea, Ingolfiellida, and Spelaeogriphacea, which are crucial for a comprehensive understanding of peracarid evolution and were incorporated in our analyses. In contrast to earlier single-gene studies (eg Jarman et al. 2000; Spears et al. 2005; Meland and Willassen 2007) and some morphological studies (eg Watling 1981) that refuted peracarid monophyly, our results firmly place Mysida within a monophyletic Peracarida and monophyletic Mysidacea, rejecting a closer relationship of Mysida and Euphausiacea.

Resolving the position of Thermosbaenacea is important for understanding the evolution of the peracarid marsupium, which is closely associated with extensive brood care and (pseudo-) direct development. Our analyses conflicted in the placement of Thermosbaenacea: Bayesian analyses using the CAT + GTR model place Thermosbaenacea as the sister group to all other Peracarida (agreeing with Siewing 1956, 1963; Richter and Scholtz 2001; Iwasa-Arai et al. 2026), while all other analytical methods recover it nested within Peracarida, either as the sister group to all nonmysidacean peracarids or as the sister to Amphipoda + Ingolfiellida. While the Amphipoda + Ingolfiellida topology was recovered in most analyses, support for this topology was reduced when the dataset was optimized for various parameters. An optimization for less missing data, increased treeness, and more variable sites (= more information content) supported the sister group relationship of Thermosbaenacea to all other Peracarida. The CAT + GTR model is assumed to best account for artifacts, such as substitutional heterogeneity and long-branch attraction (Lartillot et al. 2009). The extremely short basal peracarid branches obtained in the CAT + GTR analyses suggest that long-branch attraction and/or similar artifacts might have caused the alternative topologies in the other analyses. Furthermore, no morphological synapomorphies are known that support a clade consisting of Thermosbaenacea and Amphipoda + Ingolfiellida or Thermosbaenacea and all other nonmysid peracarids. These placements of Thermosbaenacea within Peracarida would imply either a loss of the oostegites (= plate-like structures on the pereiopods forming the marsupium) in Thermosbaenacea, or alternatively, a thrice independent evolution of the structurally complex oostegal marsupium (in Mysidacea, Amphipoda + Ingolfiellida, and Mancoida + Mictacea, respectively). If Thermosbaenacea is the sister group to all other Peracarida, we have to consider that direct development evolved independently in Thermosbaenacea and all other nonmysidacean peracarids, given that Mysidacea exhibit pseudodirect development. The independent acquisition of direct development and the marsupium is further supported by the uniqueness of the thermosbaenacean brooding system, involving egg sacks attached to the sixth thoracopods (Zilch 1972), in which early embryos are shortly carried prior to their relocation to the dorsal brood chamber (Olesen et al. 2015). Furthermore, the presence of an embryonic ring of 19 ectoteloblasts in *T. mirabilis* (Zilch 1972) is a feature found consistently in nonperacaridan malacostracans. In contrast, all other peracarids show on their germ band a varying number of ectoteloblasts arranged in a row (Scholtz 2000), except for amphipods, which lack ectoteloblasts entirely (Scholtz and Wolff 2002). Thus, embryology and the unique brooding system as well as our Bayesian analyses strongly argue for a sister group relationship of Thermosbaenacea to all other Peracarida, as was also recovered in a recent study by Iwasa-Arai et al. (2026). Notably, after our analyses were completed, the genome of *Tethysbaena scabra* was published (Pons et al. 2025). Future studies incorporating additional genomic data from Thermosbaenacea may help stabilize their position.

Building on this evolutionary change in developmental mode, our phylogenetic results also reveal a corresponding pattern in molecular evolution: Peracarid lineages consistently exhibit significantly longer branch lengths and thus higher rates of molecular evolution compared to other malacostracans. The overall longer branches for Peracarida are not restricted to our study, but can also be observed in numerous other phylogenetic analyses based on a variety of molecular markers (Spears et al. 2005; Meland and Willassen 2007; Schwentner et al. 2018; Bernot et al. 2023; Yu et al. 2024; Dreyer et al. 2025). Although the underlying processes are unknown, it appears likely that the accelerated rate of molecular evolution is intrinsically linked to the evolutionary shift in their reproductive strategy and associated changes in population genetic properties. The evolution of the marsupium reduced the number of offspring per brood (a shift toward a more strongly K-selected strategy compared to other Malacostraca) and resulted in the loss of a dispersive larval stage. The latter reduced the overall dispersal capabilities of Peracarida and may promote population fragmentation and inbreeding. All these factors affect microevolutionary processes, including a reduction of the effective population size (*N*_e_), and reduced *N*_e_ has been shown to enhance the effects of genetic drift and increase the fixation rate of slightly deleterious mutations, thereby increasing the overall rate of molecular evolution (Bromham 2011; Lanfear et al. 2014). Foltz (2003) demonstrated that marine invertebrates lacking pelagic larvae had higher levels of nonsynonymous substitutions (ie higher rates of molecular evolution at the AA level as observed for Peracarida) than those with pelagic larvae. Supporting this interpretation, the lowest relative substitution rates within Peracarida occur in Mysidacea, which retain pseudodirect development that likely represents an intermediate stage to true direct development in Amphipoda, Ingolfiellida, and Mancoida. We further demonstrate that the trend of accelerated molecular evolution in Peracarida persists at the order level, with Lophogastrida representing the only group that did not exhibit significantly longer branch lengths relative to the other Malacostraca. However, these results should be interpreted with caution, as some groups, including Lophogastrida, are represented by a limited number of taxa in our dataset. Consequently, we cannot distinguish whether the lack of significant differences reflects statistical artifacts associated with low sample size or genuine biological patterns. It is possible that these lineages exhibit lower evolutionary or diversification rates, consistent with their comparatively low species richness. Such life-history and/or ecological effects on the molecular rate of evolution have been largely studied for mammals (reviewed in Bromham 2011; Lanfear et al. 2014), and Malacostraca, especially Peracarida, may be another well-suited example for future studies in this matter. Increased taxon sampling will be necessary to robustly assess whether these patterns persist and to evaluate whether similar associations are evident within individual groups.

Beyond these patterns of molecular evolution, our phylogenomic results also provide clarity on several deep phylogenetic relationships. Among these, we found a notable congruence with major morphological hypotheses, recovering a well-supported Mancoida clade, including Mictacea (see also Iwasa-Arai et al. 2026). The robustness of this clade is further reinforced by comprehensive morphological data (Richter and Scholtz 2001; Wirkner and Richter 2010; Grams et al. 2025), which placed Bochusacea (missing in our study) within this clade as well (Wilson 2009; Grams et al. 2025). In fact, manca larvae have been confirmed for Mictacea (Bowman & Iliffe 1985) and (so far) one species of Bochusacea (Guțu et al. 1998). Due to the strong genomic evidence as well as shared developmental and morphological traits, we propose for Mictacea to be classified as part of Mancoida. A synapomorphy of Mancoida is an early embryo with dorsally flexed caudal papillae (Richter and Scholtz 2001; Olesen et al. 2015). As for the exact position of Mictacea within Mancoida, our analyses were not consistent. Mictacea was placed either as the sister group to Isopoda + Cumacea + Tanaidacea or, alternatively, as the sister group to the Spelaeogriphacea, hence forming the clade Cosinzeneacea proposed by Guțu (1998), with Cosinzeneacea as the sister group to the remaining Mancoida. We further endorse the inclusion of Bochusacea in Mancoida based on morphological, developmental, and prior cladistic evidence, even though our analyses (due to lacking representatives) cannot provide support for this proposition.

According to the classification of Lowry and Myers (2017), Ingolfiellidea was placed outside of Amphipoda and raised to order status. Our analyses recovered these two taxa as sister groups. Hence, both classifying them as separate orders and uniting them within a broader concept of Amphipoda are justified interpretations. Some morphological studies supported a sister-group relationship between the species-rich taxa Isopoda and Amphipoda (at that time including Ingolfiellida), based on putative synapomorphies, such as sessile eyes, the reduction of the carapace, and the reduction of exopods on the thoracopods (eg Wagner 1994; Wills 1998; Poore 2005; Grams et al. 2025). However, our recovery of Mancoida is in line with other morphological and molecular studies (eg Richter and Scholtz 2001; Wilson 2009; Wirkner and Richter 2010; Spears et al. 2005; Meland and Willassen 2007; Schwentner et al. 2018; Höpel et al. 2022; Bernot et al. 2023; Iwasa-Arai et al. 2026) rejecting a closer relationship of Amphipoda and Isopoda.

Within Isopoda, our analyses consistently recovered the monophyly of the major suborders Valvifera, Oniscidea, and Asellota, which are well supported by molecular and morphological work (Wägele 1989; Wetzer et al. 1997; Thomas Thorpe 2024; Figs. S5 and S6). Furthermore, our findings strongly support the “CLVS” clade (Cymothooidea, Limnoriidea, Valvifera, Sphaeromatidea) proposed by Thomas Thorpe (2024). Within this clade, our results suggest a sister-group relationship between Valvifera and Seroloidea (Sphaeromatidea), rendering Sphaeromatidea paraphyletic (see also Wetzer et al. 2013; Lins et al. 2017; Thomas Thorpe 2024). Notably, our analysis places Anthuroidea (Cymothoida) as the sister group to the entire CLVS clade, a finding that would render Cymothoida paraphyletic and suggest that Anthuroidea represents a distinct lineage. While current taxonomy places Gnathiidae within Cymothoida, our results suggest a closer relationship between Gnathiidae and Epicaridea (see also Brusca and Wilson 1991). The entire CLVS clade (excluding Gnathiidae) forms the sister group to Oniscidea, with Epicaridea, Gnathiidae, and Asellota splitting off earlier. However, one limitation of this analysis was the absence of important isopod lineages, including Phreatoicidea. Our findings, congruent with Thomas Thorpe (2024), demonstrate that parasitism evolved independently in Cymothoida and in the Epicaridea–Gnathiidae clade. The sister-group relationship between Epicaridea and Gnathiidae implies a hematophagous common ancestor, contradicting the hypothesis of an epicaridean origin from fish parasites (Dreyer and Wägele 2001). While these results clarify major evolutionary transitions, a broader sampling of parasitic taxa (eg Cirolanidae and Corallanidae) remains essential for a comprehensive understanding.

We present a well-supported phylogeny for Tanaidacea (Fig. S8) that corroborates the superfamilial relationships with current taxonomy: Apseudoidea represents the sister taxon to all others, followed by the successive divergences of Paratanaoidea and a clade comprising the sister groups Tanaidoidea and Neotanaoidea. This topology is fully congruent with earlier findings of Drumm (2010), based on three markers (COI, H3, and 28S), and is further corroborated by the transcriptome-based phylogeny of Kakui et al. (2021), from which we sourced the majority of our tanaidacean sequences.

Our analysis largely confirms the monophyly of major cumacean families (Fig. S4; see also Gerken et al. 2022), though we recover Bodotriidae as paraphyletic. The position of Diastylidae as the sister group to all other cumaceans included is strongly supported (Haye et al. 2004; Luque and Gerken 2019; Gerken et al. 2022). However, our results suggest a key revision of the internal relationships of the latter clade: We find robust support for a closer relationship between Bodotriidae and Nannastacidae, with Leuconidae representing their sister group. This stands in contrast to the topology of Gerken et al. (2022), which suggested Leuconidae and Nannastacidae as sister taxa. Our current limited taxon sampling highlights a need for future comprehensive phylogenies with denser sampling, including the missing cumacean families Ceratocumatidae, Gynodiastylidae, Lampropidae, and Pseudocumatidae, to fully resolve cumacean evolutionary relationships.

Our results highlight the unresolved backbone of amphipod phylogeny, contrasting with current taxonomic frameworks (Figs. S2 and S3; Lowry and Myers 2017). Deep relationships, including the placement of Hyperiidea and the paraphyletic clades of Senticaudata and Amphilochidea, remained unstable (see also Copilaş-Ciocianu et al. 2020; Bernot et al. 2023; Dreyer et al. 2025; Iwasa-Arai et al. 2026). The paraphyly of Hyaloidea and Gammaroidea, as well as the variable positions of lysianassoid taxa, imply that current diagnostic morphological characters may not reliably reflect evolutionary history in these groups. Overall, our findings emphasize the need for an expanded taxonomic sampling to stabilize deep nodes of the Amphipoda.

We recover Mysida as the sister group to Lophogastrida and Stygiomysida, thereby supporting a monophyletic Mysidacea (Fig. S7; see also Höpel et al. 2022; Bernot et al. 2023; Grams et al. 2025). Mysida comprises two families, differentiated by the presence (Mysidae) or absence (Petalophthalmidae) of a statocyst in the uropods (Meland et al. 2015). Our phylogeny supports this classification with *Hansenomysis* sp. (Petalophthalmidae) recovered as the sister group to all other sampled mysids, representing five of the ten subfamilies in the family Mysidae (Meland and Willassen 2007; Meland et al. 2015; Kou et al. 2025).

In a broader malacostracan perspective, the phylogenetic placement of Stomatopoda has been a persistent challenge. Most of our analyses provide robust support for positioning Stomatopoda as the sister group to Caridoida (all other Eumalacostraca).

This placement is consistent with results from a subset of analyses in recent phylogenomic studies (Schwentner et al. 2018; Bernot et al. 2023) as well as previous morphological studies (eg Siewing 1956; Richter and Scholtz 2001; Wirkner and Richter 2010). Alternatively, a closer relationship between Stomatopoda and Eucarida and/or Syncarida has been proposed (eg Höpel et al. 2022; Bernot et al. 2023), which we recovered in very few analyses (mainly in ASTRAL analyses). Relationships among other major lineages remain contentious. The monophyly of Eucarida (Decapoda + Euphausiacea) is notably unresolved, as Euphausiacea forms a clade either with Decapoda or with Anaspidacea. Syncarida (Bathynellacea + Anaspidacea) is consistently recovered as polyphyletic, similar to the findings of Bernot et al. (2023), though the unstable placement of *Allobathynella bangokensis* (Bathynellacea) underscores lingering uncertainty in syncarid interrelationships.

This phylogenomic study provides a significantly resolved phylogenetic framework for Peracarida, one of the most species-rich taxa within crustaceans, reconciling long-standing conflicts between morphological and molecular data. Our robust support for a monophyletic Peracarida, with Stomatopoda as the sister group to all other Eumalacostraca (Malacostraca except Leptostraca), offers a stable foundation for understanding the evolution of this ecologically crucial group. We demonstrate that molecular evolutionary rates are accelerated across peracarid lineages and suggest that key peracarid innovations—the marsupium and direct development—drove a shift in reproductive strategy (reducing dispersal and effective population size). These innovations offer a potential mechanistic explanation, linking a profound change in developmental mode to its long-term genomic consequences. While deep relationships within some taxa, such as Amphipoda, remain challenging to resolve, our results provide a backbone across Isopoda, Tanaidacea, Cumacea, and Mysidacea. This work underscores the necessity of combining dense taxon sampling with phylogenomic data to test morphological hypotheses and reconstruct the evolutionary history of complex radiations. This study also charts a course for future research, with critical next steps including incorporating more Thermosbaenacea and representatives of Bochusacea to further test the relationships of Peracarida, especially concerning the contentious placement of Thermosbaenacea.

## Materials and methods

### Sample collection and sequencing

Taxon sampling comprised 156 taxa represented by annotated genomes or transcriptomes. Of these, 43 publicly available transcriptomes were downloaded from the NCBI Sequence Read Archive (SRA) as raw reads, 43 assembled transcriptomes were downloaded from NCBI TSA, and 6 genome annotations were downloaded from NCBI Genome (Table S1). Publicly available data were supplemented with 64 transcriptomes that were newly sequenced for this study. Outgroup taxon sampling was performed to span all extant nonperacarid malacostracan lineages, including species spanning the phylogenetic diversity of each clade. Leptostraca (*Nebalia* spp.) was used to root the trees.

For libraries generated by the Kocot lab (Table S1), RNA was extracted from RNAlater or cryopreserved specimens using the Omega Bio-Tek RNA MicroElute Kit. RNA concentration was measured using a Thermo Fisher Qubit 3.0 fluorometer with the RNA High Sensitivity kit, purity was assessed by measuring the 260/280 nm absorbance ratio using a Thermo Fisher Nanodrop Lite, and integrity was confirmed using a 1% SB agarose gel or an Agilent Fragment Analyzer. Complementary DNA (cDNA) synthesis was performed using 1 ng of total RNA using the Clontech SMART-Seq HT kit. An Illumina sequencing library was then prepared using either the Takara SMART-Seq HT Plus kit with 2 to 10 ng of cDNA with a 60 min enzymatic fragmentation step and the number of PCR cycles recommended by the manufacturer or the Nextera XT kit with 0.125 ng of cDNA. cDNA and Sequencing library molecular weight distribution and concentration were assessed using an Agilent Fragment Analyzer with the NGS Fragment Kit (1 to 6,000 bp). Resulting libraries were pooled and sent to Azenta (Plainfield, NJ, USA) for sequencing on either an Illumina NovaSeq 6000 S4 flowcell or an Illumina NovaSeq X Plus 25B flow cell with 2 × 150 bp PE reads. For libraries generated by the Schwentner lab, whole specimens were preserved in RNAlater, stored at −80 °C, and submitted to Macrogen (Amsterdam, Netherlands) for RNA extraction, cDNA synthesis, and sequencing library preparation using the TruSeq Stranded mRNA Library Prep kit. In some cases, the digestive tract was removed before preservation. Sequencing was performed on an Illumina NovaSeq 6000 S4 flow cell with 2 × 150 bp PE reads.

### Transcriptome assembly and phylogenomic dataset construction

Raw reads were assembled into transcripts using *Trinity v2.132 or v2.10.0* (Grabherr et al. 2011). Assembly completeness was assessed by using *BUSCO v5.2.2* (Manni et al. 2021) against the *arthropoda_odb10* database; assemblies with low *BUSCO* scores were excluded unless critical for taxonomic representation.

Coding regions were predicted using *TransDecoder v5.7.1* (Haas, BJ. https://github.com/TransDecoder/TransDecoder), initially retaining all open reading frames (ORFs) ≥ 100 AA. To maximize sensitivity for functionally significant ORFs, we validated these predictions through (i) *DIAMOND v2.1.9* (Buchfink et al. 2021) similarity searches against the *UniProtKB/Swiss-Prot* database and (ii) domain detection using *HMMER v3.4* (Zhang and Wood 2003). The resulting BLAST and Pfam outputs were integrated through TransDecoder.Predict, with applied filters to retain only the single best coding region prediction per transcript meeting a minimum length threshold of 500 AA. Identifying orthologous genes is one of the most crucial and contentious steps in phylogenomic studies. To mitigate this issue, we employed two different strategies to identify orthologous genes, one tree-based approach using *OrthoFinder* and one based on BUSCO genes, and generated and analyzed separate data matrices for each. For the ORTHO datasets, orthogroups were inferred using *OrthoFinder v2.5.5* (Emms and Kelly 2019). To mitigate biases from gene duplication or misassembly, orthogroups containing >10× transcripts per taxon were excluded. Fragmentary sequences <100 AA were discarded. For the BUSCO datasets, single-copy orthologs identified by BUSCO were extracted using a custom Python script (BUSCO_retrieve_genes.py) to create the first phylogenomic dataset (= BUSCO).

Retained sequences from both datasets were aligned with *MAFFT v7.487* (Katoh and Standley 2013). The ORTHO dataset alignments were further cleaned with *HmmCleaner* (Di Franco et al. 2019) to remove potentially mistranslated sequence regions. Alignments sampled for <50% of taxa after this cleaning were discarded to minimize overall missing data. Gene trees were reconstructed for each orthogroup using *FastTree v2.2* (Price et al. 2009, 2010) with the -gamma model. Trees were processed with *PhyloPyPruner* (https://gitlab.com/fethalen/phylopypruner) to remove paralogous sequences using the settings –min-len 100 –min-support 0.75 –mask pdist –trim-lb 6 –trim-divergent 0.50 –min-pdist 2e-8 –prune LS. For each orthogroup, only one sequence was retained per taxon according to the “Largest Subtree” algorithm, and resulting orthogroups with fewer than 75% of the taxa sampled were discarded.

The BUSCO and ORTHO alignments were trimmed using *ClipKIT v2.3.0* (Steenwyk et al. 2020) to remove sites where gaps occurred in ≤50% of sequences. To filter the trimmed alignments again using *PhlyoPyPruner* (75% occupancy), gene trees were reconstructed using *FastTree v2.2* as described above. We retained 673 loci from the BUSCO dataset and 511 from the ORTHO dataset for downstream analyses.

From these datasets, two additional datasets were created by filtering for the most informative loci. Using *genesortR* (Mongiardino Koch 2021), an R-based tool that quantifies phylogenetic utility based on average pairwise patristic distance, compositional heterogeneity, level of saturation, RTT variance, Robinson–Foulds similarity to a target topology, average bootstrap support, and the proportion of variable sites, all loci were ranked by their evolutionary informativeness. For each dataset, we retained only the top 50% highest-ranking loci, resulting in a BUSCO-50% dataset (337 loci) and an ORTHO-50% dataset (255 loci).

### Phylogenetic analyses

We subjected all four datasets to six different phylogenetic approaches. We performed three ML analyses in *IQ-TREE 2 v1.5.0* (Minh et al. 2020): (i) standard inference using the ModelFinder-embedded MFP algorithm; (ii) the GHOST framework (Crotty et al. 2020), which partitions sites into multiple rate categories; and (iii) the site-heterogeneous PMSF approximation (Wang et al. 2018), using posterior mean site frequencies derived from a profile mixture model (LG + C20 + F + G). Branch support for all three analyses was assessed through 1,000 ultrafast bootstrap replicates (UFBoot). We further implemented a coalescence-based approach using ASTRAL-III v5.7.7 (Zhang et al. 2018) to account for incomplete lineage sorting. Input gene trees were generated in *IQ-TREE 2* under the MFP model with 1,000 UFBoot replicates to assess branch support. To mitigate potential artifacts from compositional heterogeneity and substitution saturation, we also implemented Dayhoff six-state recoding, which clusters AA into six functionally similar groups based on substitution probabilities from the Dayhoff/Pam250 matrix (Dayhoff et al. 1978). The recoded alignments were then analyzed in *RAxML-NG v8.2.12* (Stamatakis 2014) using the MULTIGAMMAI model with 1,000 UFBoot replicates to assess branch support. Finally, we conducted a MP analysis using *wTNT v.1.6 2025-06-22* (Goloboff and Morales 2023) using the “PhylogenomicSearch.run” script (Torres et al. 2022) with the command “run PhylogenomicSearch.run input DATASET_NAME.tnt search nt level 4 hits 2 output newick;” and calculating bootstrap support with the “PhylogenomicSupport.run” script (Torres et al. 2022) with the command “run PhylogenomicSupport.run input DATASET_NAME.tnt reftree REFERENCETREE_NAME.tre sites boot replic 1000 search nt level 1 output newick;”.

Representing the most complex model used in our study, BI was implemented using *PhyloBayes MPI v1.9* (Lartillot et al. 2013) under the CAT-GTR model. Due to the method's computational demands, we restricted this analysis to the 50% datasets (BUSCO-50% and ORTHO-50%) with a strategically reduced taxon set (*n* = 62), preserving all major clades. For the ORTHO-50% dataset, three independent chains were run for 10,069 generations each, with sampling every 10th generation after discarding 2,500 generations as burn-in. The BUSCO-50% dataset was analyzed with two chains of 10,202 generations under identical sampling and burn-in parameters. We discarded the first 2,500 trees from each chain as burn-in and calculated a 50% majority rule consensus tree from the remaining trees from each chain for each dataset. For each dataset, the individual chains did not converge after more than 10,000 generations. Since there were no signs of convergence even after more than 10,000 generations each (maxdiff: 1), the cutoff was set here.

As the position of Thermosbaenacea differed strongly between the PhyloBayes CAT + GTR and all other analyses, we employed two approaches to better understand the underlying causes. First, we investigated if any of these alternative topologies is better supported by sCF or gCF. These were computed exemplarily for the MFP and PhyloBayes analyses of the ORTHO-50% dataset in *IQ-TREE 2* using the -t option, as these represent two alternative positions of Thermosbaenacea, thereby estimating the proportion of gene trees and informative sites supporting each branch and assessing concordance and conflict among loci. Second, we optimized and subsampled one of our datasets for several gene properties, to assess how these affect the phylogenetic position of Thermosbaenacea. We used 12 of the properties included in *genesortR* (we only omitted Robinson–Foulds similarity as this is biased by an input topology) to subsample the BUSCO dataset. For each of 12 locus properties, we extracted the loci with the 50% highest and lowest values, respectively, resulting in 24 subsets. These properties included average bootstrap support, alignment length, occupancy, treeness (ie the proportion of branch lengths attributable to internal branches), average patristic distance, proportion of missing data, rate of evolution, compositional heterogeneity [estimated using relative composition frequency variability {RCFV}], RTT variance, substitution saturation level, total tree length, and the proportion of variable sites (Mongiardino Koch 2021). It should be noted that higher values are not necessarily “better”; for example, for the proportion of missing data, lower values are probably better. The resulting subsampled alignments were then subjected to phylogenetic analysis using the PMSF method in *IQ-TREE 2* as described above.

### Evolutionary rate calculations

To quantify evolutionary rates across Malacostraca, we calculated RTT branch lengths for each terminal in all phylogenetic analyses except TNT, using custom Python and Bash scripts. All trees were rooted with Leptostraca as the outgroup to standardize distance measurements. We excluded *Hyperia* sp. due to its exceptionally long branches, which could disproportionately influence RTT comparisons. Stomatopoda were also excluded from the analysis to restrict comparisons to Peracarida and their putative sister group (Decapoda + Euphausiacea + Anaspidacea + Bathynellacea). Our final dataset included 3,095 RTT observations across 22 trees. Because phylogenetic trees were used as replicates, each taxon contributed to 22 RTT observations in our models. We used linear mixed models (LMM) using the Ime4 and ImerTest R packages (Bates et al. 2015; Kuznetsova et al. 2017) to evaluate RTT differences between Peracarida and all nonperacarids (model 1) and RTT differences among Peracarida orders and all nonperacarids (model 2). Prior to running the analyses, RTT were log transformed to fit LMM assumptions. In these models, log-transformed RTT represents the response variable, taxonomic groups represent fixed effects (model 1: Peracarida, non-Peracarida; model: Amphipoda, Cumacea, Isopoda, Tanaidacea, Mysida, Spelaeogriphacea, Thermosbaenacea, Ingolfiellida, Stygiomysida, Lophogastrida, non-Peracarida), and tree and species represent random intercepts. Random intercepts were included to account for different branch-length scales resulting from different trees. For model 1, estimated marginal means were used for pairwise contrasts between Peracarida and non-Peracarida. For model 2, estimated marginal means were used for Tukey-adjusted multiple pairwise comparisons among taxonomic groups. Mean taxonomic group differences with 95% CIs were visualized using forest plots of RTT ratios using the non-Peracarida as a reference. For visualization purposes only, we also created a violin plot using every entry for the analyses. In this case, data was normalized per tree by calculating RTT_species_/mean non-Peracarida RTT from the same tree. This approach allowed all species to have their RTT compared to the outgroup from the same tree at the same scale and facilitating visualization. Branch lengths in all ASTRAL analyses were distinctly lower (Fig. S35), probably because branch lengths in ASTRAL are measures of coalescence units and not substitutions per site as in all other analyses. Because branch lengths stemming ASTRAL analyses will affect the inferred evolutionary rate differences, the respective analyses were run once with and once without the ASTRAL-based branch lengths.

## Supplementary Material

msag178_Supplementary_Data

## Data Availability

Sample information and sequence data for de novo transcriptomes are available in NCBI (BioProject P RJNA1480557; SAMN61033789–SAMN61033852; SRR39399494–SRR39399557). Additional materials, including phylogenetic alignments, tree files, branch-length metrics, and custom analysis scripts (Bash/Python), have been deposited in our data repository (https://doi.org/10.57756/xanenh).
