# Supplementary material for: Phylogenomics supports monophyly of marsupial crustaceans: a journey to direct development
Source: Mol Biol Evol. 2026 Aug 13;43(8):msag178. doi: 10.1093/molbev/msag178 (PMC13472170; doi:10.1093/molbev/msag178)
Supplement: Supplementary file 1 [file msag178_supplementary_data.zip › S15_BUSCO50_MFP.pdf]

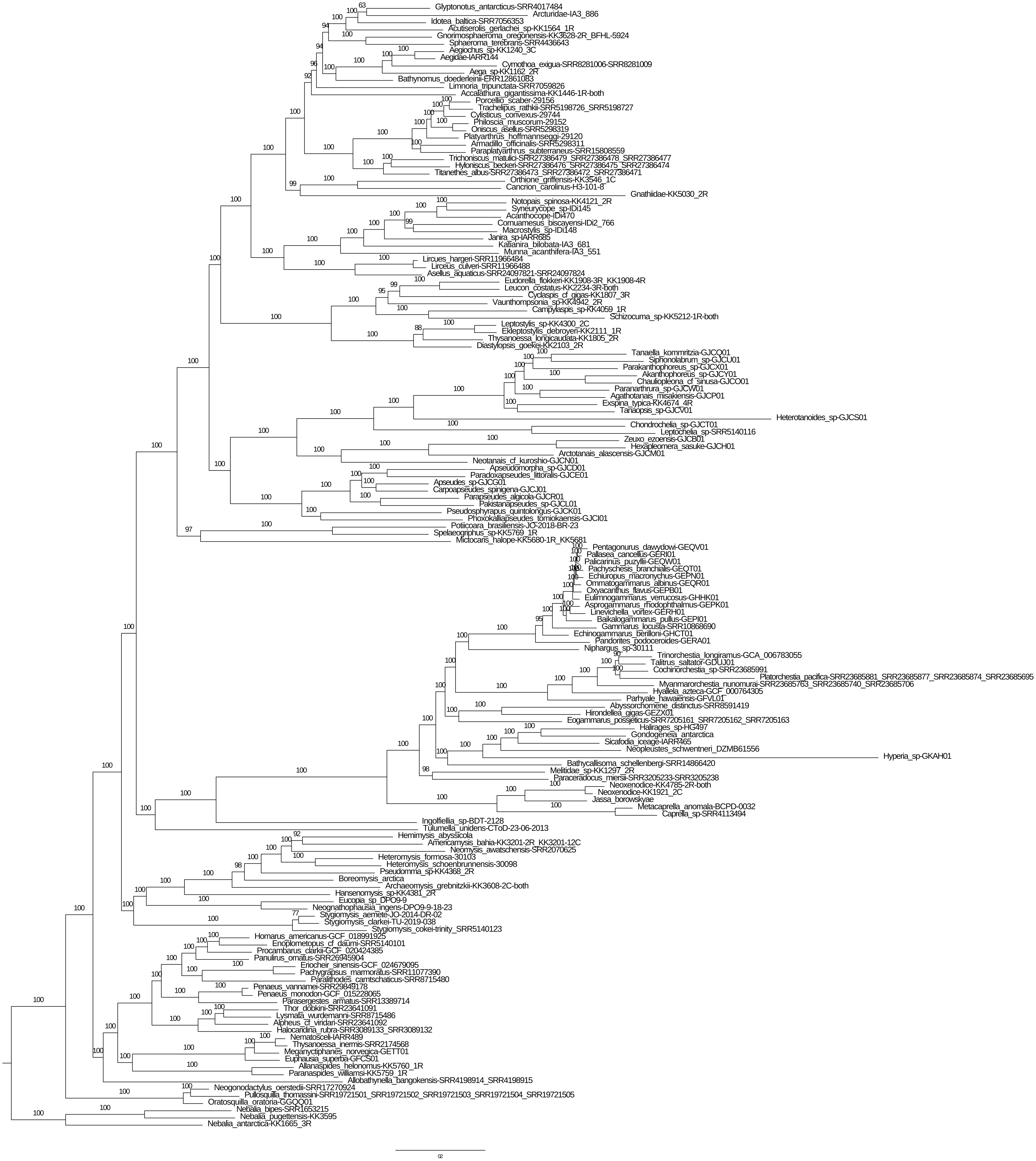
Glyptonotus_antarcticus-SRR4017484
Arcturidae-IA3_886
Idotea_baltica-SRR7056353
Acutiserolis_gerlachei_sp-KK1564_1R
Gnorimosphaeroma_oregonensis-KK3628-2R_BFHL-5924
Sphaeroma_terebrans-SRR4436643
Aegiochus_sp-KK1240_3C
Aegidae-IARR144
Cymothoa_exigua-SRR8281006-SRR8281009
Aega_sp-KK1162_2R
Bathynomus_doederleinii-ERR12861083
Limnoria_tripunctata-SRR7059826
Accalathura_gigantissima-KK1446-1R-both
Porcellio_scaber-29156
Trachelipus_rathkii-SRR5198726_SRR5198727
Cylisticus_convexus-29744
Philoscia_muscorum-29152
Oniscus_asellus-SRR5298319
Platyarthrus_hoffmannseggi-29120
Armadillo_officinalis-SRR5298311
Paraplatyarthrus_subterraneus-SRR15808559
Trichoniscus_matulici-SRR27386479_SRR27386478_SRR27386477
Hyloniscus_beckeri-SRR27386476_SRR27386475_SRR27386474
Titanethes_albus-SRR27386473_SRR27386472_SRR27386471
Orthione_griffensis-KK3546_1C
Cancrion_carolinus-H3-101-8
Gnathiidae-KK5030_2R
Notopais_spinosa-KK4121_2R
Syneurycope_sp-IDi145
Acanthocope-IDi470
Cornuamesus_biscayensis-IDi2_766
Macrostylis_sp-IDi148
Janira_sp-IARR685
Katianira_bilobata-IA3_681
Munna_acanthifera-IA3_551
Lirceus_hargeri-SRR11966484
Lirceus_culveri-SRR11966488
Asellus_aquaticus-SRR24097821-SRR24097824
Eudorella_flokkeri-KK1908-3R_KK1908-4R
Leucon_costatus-KK2234-3R-both
Cyclaspis_cf_gigas-KK1807_3R
Vaunthompsonia_sp-KK4942_2R
Campylaspis_sp-KK4059_1R
Schizocuma_sp-KK5212-1R-both
Leptostylis_sp-KK4300_2C
Ekleptostylis_debroyeri-KK2111_1R
Thysanoessa_longicaudata-KK1805_2R
Diastylopsis_goekei-KK2103_2R
Tanaella_kommritzia-GJCQ01
Siphonolabrum_sp-GJCU01
Parakanthophoreus_sp-GJCX01
Akanthophoreus_sp-GJCY01
Chauliopleona_cf_sinusa-GJCO01
Paranarthrura_sp-GJCW01
Agathotanais_misakiensis-GJCP01
Exspina_typica-KK4674_4R
Tanaopsis_sp-GJCV01
Heterotanoides_sp-GJCS01
Chondrochelia_sp-GJCT01
Leptochelia_sp-SRR5140116
Zeuxo_ezoensis-GJCB01
Hexapleomera_sasuke-GJCH01
Arctotanais_alascensis-GJCM01
Neotanais_cf_kuroshio-GJCN01
Apseudomorpha_sp-GJCD01
Paradoxapseudes_littoralis-GJCE01
Apseudes_sp-GJCG01
Carpoapseudes_spinigena-GJCJ01
Parapseudes_algicola-GJCR01
Pakistanapseudes_sp-GJCL01
Pseudosphyrapus_quintolongus-GJCK01
Phoxokalliapseudes_tomiokaensis-GJCI01
Potiicoara_brasiliensis-JO-2018-BR-23
Spelaeogriphus_sp-KK5769_1R
Mictocaris_halope-KK5680-1R_KK5681
Pentagonurus_dawydowi-GEQV01
Pallasea_cancellus-GERI01
Palicarinus_puzyllii-GEQW01
Pachyschesis_branchialis-GEQT01
Echiuropus_macronychus-GEPN01
Ommatogammarus_albinus-GEQR01
Oxyacanthus_flavus-GEPB01
Eulimnogammarus_verrucosus-GHHK01
Asprogammarus_rhodophthalmus-GEPK01
Linevichella_vortex-GERH01
Baikalogammarus_pullus-GEPI01
Gammarus_locusta-SRR10868690
Echinogammarus_berilloni-GHCT01
Pandorites_podoceroides-GERA01
Niphargus_sp-30111
Trinorchestia_longiramus-GCA_006783055
Talitrus_saltator-GDUJ01
Cochinorchestia_sp-SRR23685991
Platorchestia_pacifica-SRR23685881_SRR23685877_SRR23685874_SRR23685695
Myanmarorchestia_nunomurai-SRR23685763_SRR23685740_SRR23685706
Hyallela_azteca-GCF_000764305
Parhyale_hawaiensis-GFVL01
Abyssorchomene_distinctus-SRR8591419
Hirondellea_gigas-GEZX01
Eogammarus_possjeticus-SRR7205161_SRR7205162_SRR7205163
Halirages_sp-HG497
Gondogeneia_antarctica
Sicafodia_iceage-IARR465
Neopleustes_schwentneri_DZMB61556
Hyperia_sp-GKAH01
Bathycallisoma_schellenbergi-SRR14866420
Melitidae_sp-KK1297_2R
Paraceradocus_miersii-SRR3205233-SRR3205238
Neoxenodice-KK4785-2R-both
Neoxenodice-KK1921_2C
Jassa_borowskyae
Metacaprella_anomala-BCPD-0032
Caprella_sp-SRR4113494
Ingolfiellia_sp-BDT-2128
Tulumella_unidens-CToD-23-06-2013
Hemimysis_abyssicola
Americamysis_bahia-KK3201-2R_KK3201-12C
Neomysis_awatschensis-SRR2070625
Heteromysis_formosa-30103
Heteromysis_schoenbrunnensis-30098
Pseudomma_sp-KK4368_2R
Boreomysis_arctica
Archaeomysis_grebnitzkii-KK3608-2C-both
Hansenomysis_sp-KK4381_2R
Eucopia_sp_DPO9-9
Neognathophausia_ingens-DPO9-9-18-23
Stygiomysis_aemete-JO-2014-DR-02
Stygiomysis_clarkei-TU-2019-038
Stygiomysis_cokei-trinity_SRR5140123
Homarus_americanus-GCF_018991925
Enoplometopus_cf_daumi-SRR5140101
Procambarus_clarkii-GCF_020424385
Panulirus_ornatus-SRR26945904
Eriocheir_sinensis-GCF_024679095
Pachygrapsus_marmoratus-SRR11077390
Paralithodes_camtschaticus-SRR8715480
Penaeus_vannamei-SRR29849178
Penaeus_monodon-GCF_015228065
Parasergestes_armatus-SRR13389714
Thor_dobkini-SRR23641091
Lysmata_wurdemanni-SRR8715486
Alpheus_cf_viridari-SRR23641092
Halocaridina_rubra-SRR3089133_SRR3089132
Nematoscelis-IARR489
Thysanoessa_inermis-SRR2174568
Meganyctiphanes_norvegica-GETT01
Euphausia_superba-GFCS01
Allanaspides_helonomus-KK5760_1R
Paranaspides_williamsi-KK5759_1R
Allobathynella_bangokensis-SRR4198914_SRR4198915
Neogonodactylus_oerstedii-SRR17270924
Pullosquilla_thomassini-SRR19721501_SRR19721502_SRR19721503_SRR19721504_SRR19721505
Oratosquilla_oratoria-GGQO01
Nebalia_bipes-SRR1653215
Nebalia_pugettensis-KK3595
Nebalia_antarctica-KK1665_3R
