# Supplementary material for: Phylogenomics supports monophyly of marsupial crustaceans: a journey to direct development
Source: Mol Biol Evol. 2026 Aug 13;43(8):msag178. doi: 10.1093/molbev/msag178 (PMC13472170; doi:10.1093/molbev/msag178)
Supplement: Supplementary file 1 [file msag178_supplementary_data.zip › S27_ORTHO_TNT.pdf]

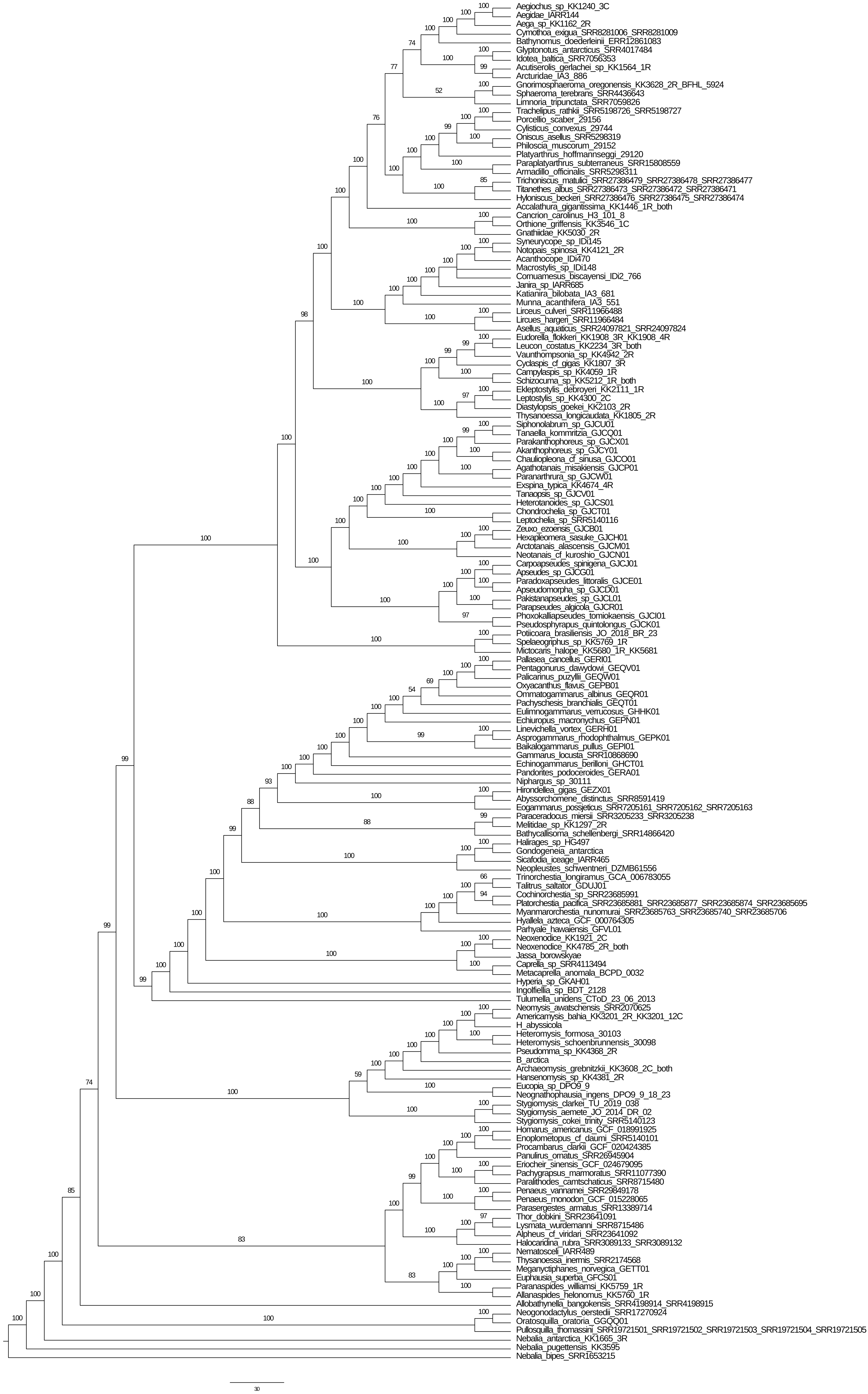
Aegiochus_sp_KK1240_3C
Aegidae_IARR144
Aega_sp_KK1162_2R
Cymothoa_exigua_SRR8281006_SRR8281009
Bathynomus_doederleinii_ERR12861083
Glyptonotus_antarcticus_SRR4017484
Idotea_baltica_SRR7056353
Acutiserolis_gerlachei_sp_KK1564_1R
Arcturidae_IA3_886
Gnorimosphaeroma_oregonensis_KK3628_2R_BFHL_5924
Sphaeroma_terebrans_SRR4436643
Limnoria_tripunctata_SRR7059826
Trachelipus_rathkii_SRR5198726_SRR5198727
Porcellio_scaber_29156
Cylisticus_convexus_29744
Oniscus_asellus_SRR5298319
Philoscia_muscorum_29152
Platyarthrus_hoffmannseggi_29120
Paraplatyarthrus_subterraneus_SRR15808559
Armadillo_officinalis_SRR5298311
Trichoniscus_matulici_SRR27386479_SRR27386478_SRR27386477
Titanethes_albus_SRR27386473_SRR27386472_SRR27386471
Hyloniscus_beckeri_SRR27386476_SRR27386475_SRR27386474
Accalathura_gigantissima_KK1446_1R_both
Cancrion_carolinus_H3_101_8
Orthione_griffensis_KK3546_1C
Gnathiidae_KK5030_2R
Syneurycope_sp_IDi145
Notopais_spinosa_KK4121_2R
Acanthocope_IDi470
Macrostylis_sp_IDi148
Cornuamesus_biscayensi_IDi2_766
Janira_sp_IARR685
Katianira_bilobata_IA3_681
Munna_acanthifera_IA3_551
Lirceus_culveri_SRR11966488
Lircues_hargeri_SRR11966484
Asellus_aquaticus_SRR24097821_SRR24097824
Eudorella_flokkeri_KK1908_3R_KK1908_4R
Leucon_costatus_KK2234_3R_both
Vaunthompsonia_sp_KK4942_2R
Cyclaspis_cf_gigas_KK1807_3R
Campylaspis_sp_KK4059_1R
Schizocuma_sp_KK5212_1R_both
Ekleptostylis_debroyeri_KK2111_1R
Leptostylis_sp_KK4300_2C
Diastylopsis_goekei_KK2103_2R
Thysanoessa_longicaudata_KK1805_2R
Siphonolabrum_sp_GJCU01
Tanaella_kommritzia_GJCQ01
Parakanthophoreus_sp_GJCX01
Akanthophoreus_sp_GJCY01
Chauliopleona_cf_sinusa_GJCO01
Agathotanais_misakiensis_GJCP01
Paranarthrura_sp_GJCW01
Exspina_typica_KK4674_4R
Tanaopsis_sp_GJCV01
Heterotanoides_sp_GJCS01
Chondrochelia_sp_GJCT01
Leptochelia_sp_SRR5140116
Zeuxo_ezoensis_GJCB01
Hexapleomera_sasuke_GJCH01
Arctotanais_alascensis_GJCM01
Neotanais_cf_kuroshio_GJCN01
Carpoapseudes_spinigena_GJCJ01
Apseudes_sp_GJCG01
Paradoxapseudes_littoralis_GJCE01
Apseudomorpha_sp_GJCD01
Pakistanapseudes_sp_GJCL01
Parapseudes_algicola_GJCR01
Phoxokalliapseudes_tomiokaensis_GJCI01
Pseudosphyrapus_quintolongus_GJCK01
Potiicoara_brasiliensis_JO_2018_BR_23
Spelaeogriphus_sp_KK5769_1R
Mictocaris_halope_KK5680_1R_KK5681
Pallasea_cancellus_GERI01
Pentagonurus_dawydowi_GEQV01
Palicarinus_puzyllii_GEQW01
Oxyacanthus_flavus_GEPB01
Ommatogammarus_albinus_GEQR01
Pachyschesis_branchialis_GEQT01
Eulimnogammarus_verrucosus_GHHK01
Echiuropus_macronychus_GEPN01
Linevichella_vortex_GERH01
Asprogammarus_rhodophthalmus_GEPK01
Baikalogammarus_pullus_GEPI01
Gammarus_locusta_SRR10868690
Echinogammarus_berilloni_GHCT01
Pandorites_podoceroides_GERA01
Niphargus_sp_30111
Hirondellea_gigas_GEZX01
Abyssorchomene_distinctus_SRR8591419
Eogammarus_possjeticus_SRR7205161_SRR7205162_SRR7205163
Paraceradocus_miersii_SRR3205233_SRR3205238
Melitidae_sp_KK1297_2R
Bathycallisoma_schellenbergi_SRR14866420
Halirages_sp_HG497
Gondogeneia_antarctica
Sicafodia_iceage_IARR465
Neopleustes_schwentneri_DZMB61556
Trinorchestia_longiramus_GCA_006783055
Talitrus_saltator_GDUJ01
Cochinorchestia_sp_SRR23685991
Platorchestia_pacifica_SRR23685881_SRR23685877_SRR23685874_SRR23685695
Myanmarorchestia_nunomurai_SRR23685763_SRR23685740_SRR23685706
Hyallela_azteca_GCF_000764305
Parhyale_hawaiensis_GFVL01
Neoxenodice_KK1921_2C
Neoxenodice_KK4785_2R_both
Jassa_borowskyae
Caprella_sp_SRR4113494
Metacaprella_anomala_BCPD_0032
Hyperia_sp_GKAH01
Ingolfiellia_sp_BDT_2128
Tulumella_unidens_CToD_23_06_2013
Neomysis_awatschensis_SRR2070625
Americamysis_bahia_KK3201_2R_KK3201_12C
H_abyssicola
Heteromysis_formosa_30103
Heteromysis_schoenbrunnensis_30098
Pseudomma_sp_KK4368_2R
B_arctica
Archaeomysis_grebnitzkii_KK3608_2C_both
Hansenomysis_sp_KK4381_2R
Eucopia_sp_DPO9_9
Neognathophausia_ingens_DPO9_9_18_23
Stygiomysis_clarkei_TU_2019_038
Stygiomysis_aemete_JO_2014_DR_02
Stygiomysis_cokei_trinity_SRR5140123
Homarus_americanus_GCF_018991925
Enoplometopus_cf_daumi_SRR5140101
Procambarus_clarkii_GCF_020424385
Panulirus_ornatus_SRR26945904
Eriocheir_sinensis_GCF_024679095
Pachygrapsus_marmoratus_SRR11077390
Paralithodes_camtschaticus_SRR8715480
Penaeus_vannamei_SRR29849178
Penaeus_monodon_GCF_015228065
Parasergestes_armatus_SRR13389714
Thor_dobkini_SRR23641091
Lysmata_wurdemanni_SRR8715486
Alpheus_cf_viridari_SRR23641092
Halocaridina_rubra_SRR3089133_SRR3089132
Nematosceli_IARR489
Thysanoessa_inermis_SRR2174568
Meganyctiphanes_norvegica_GETT01
Euphausia_superba_GFCS01
Paranaspides_williamsi_KK5759_1R
Allanaspides_helonomus_KK5760_1R
Allobathynella_bangokensis_SRR4198914_SRR4198915
Neogonodactylus_oerstedii_SRR17270924
Oratosquilla_oratoria_GGQQ01
Pullosquilla_thomassini_SRR19721501_SRR19721502_SRR19721503_SRR19721504_SRR19721505
Nebalia_antarctica_KK1665_3R
Nebalia_pugettensis_KK3595
Nebalia_bipes_SRR1653215
30
