# Supplementary material for: Phylogenomics supports monophyly of marsupial crustaceans: a journey to direct development
Source: Mol Biol Evol. 2026 Aug 13;43(8):msag178. doi: 10.1093/molbev/msag178 (PMC13472170; doi:10.1093/molbev/msag178)
Supplement: Supplementary file 1 [file msag178_supplementary_data.zip › S33_ORTHO50_TNT.pdf]

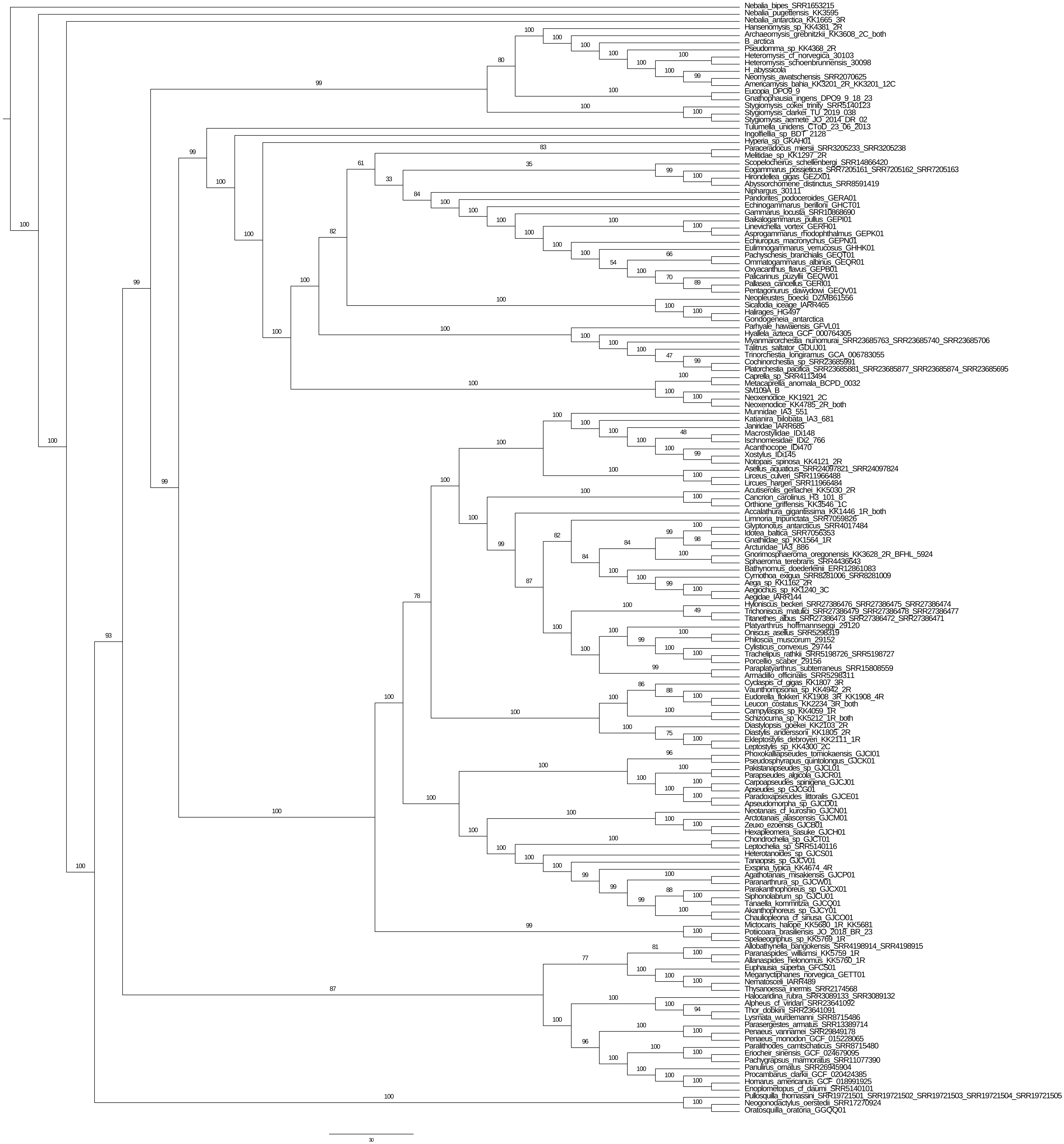
Nebalia_bipes_SRR1653215
Nebalia_pugettensis_KK3595
Nebalia_antarctica_KK1665_3R
Hansenomysis_sp_KK4381_2R
Archaeomysis_grebnitzkii_KK3608_2C_both
B_arctica
Pseudomma_sp_KK4368_2R
Heteromysis_cf_norvegica_30103
Heteromysis_schoenbrunnensis_30098
H_abyssicola
Neomysis_awatschensis_SRR2070625
Americamysis_bahia_KK3201_2R_KK3201_12C
Eucopia_DPO9_9
Gnathophausia_ingens_DPO9_9_18_23
Stygiomysis_cokei_trinity_SRR5140123
Stygiomysis_clarkei_TU_2019_038
Stygiomysis_aemete_JO_2014_DR_02
Tulumella_unidens_CToD_23_06_2013
Ingolfiellia_sp_BDT_2128
Hyperia_sp_GKAH01
Paraceradocus_miersii_SRR3205233_SRR3205238
Melitidae_sp_KK1297_2R
Scopelocheirus_schellenbergi_SRR14866420
Eogammarus_possjeticus_SRR7205161_SRR7205162_SRR7205163
Hirondellea_gigas_GEZX01
Abyssorchomene_distinctus_SRR8591419
Niphargus_30111
Pandorites_podoceroides_GERA01
Echinogammarus_berilloni_GHCT01
Gammarus_locusta_SRR10868690
Baikalogammarus_pullus_GEPI01
Linevichella_vortex_GERH01
Asprogammarus_rhodophthalmus_GEPK01
Echiuropus_macronychus_GEPN01
Eulimnogammarus_verrucosus_GHHK01
Pachyschesis_branchialis_GEQT01
Ommatogammarus_albinus_GEQR01
Oxyacanthus_flavus_GEPB01
Palicarinus_puzyllii_GEQW01
Pallasea_cancellus_GERI01
Pentagonurus_dawydowi_GEQV01
Neopleustes_boecki_DZMB61556
Sicafodia_iceage_IARR465
Halirages_HG497
Gondogeneia_antarctica
Parhyale_hawaiensis_GFVL01
Hyallela_azteca_GCF_000764305
Myanmarorchestia_nunomurai_SRR23685763_SRR23685740_SRR23685706
Talitrus_saltator_GDUJ01
Trinorchestia_longiramus_GCA_006783055
Cochinorchestia_sp_SRR23685991
Platorchestia_pacifica_SRR23685881_SRR23685877_SRR23685874_SRR23685695
Caprella_sp_SRR4113494
Metacaprella_anomala_BCPD_0032
SM109A_B
Neoxenodice_KK1921_2C
Neoxenodice_KK4785_2R_both
Munnidae_IA3_551
Katianira_bilobata_IA3_681
Janiridae_IARR685
Macrostylidae_IDi148
Ischnomesidae_IDi2_766
Acanthocope_IDi470
Xostylus_IDi145
Notopais_spinosa_KK4121_2R
Asellus_aquaticus_SRR24097821_SRR24097824
Lirceus_culveri_SRR11966488
Lircues_hargeri_SRR11966484
Acutiserolis_gerlachei_KK5030_2R
Cancrion_carolinus_H3_101_8
Orthione_griffensis_KK3546_1C
Accalathura_gigantissima_KK1446_1R_both
Limnoria_tripunctata_SRR7059826
Glyptonotus_antarcticus_SRR4017484
Idotea_baltica_SRR7056353
Gnathiidae_sp_KK1564_1R
Arcturidae_IA3_886
Gnorimosphaeroma_oregonensis_KK3628_2R_BFHL_5924
Sphaeroma_terebrans_SRR4436643
Bathynomus_doederleinii_ERR12861083
Cymothoa_exigua_SRR8281006_SRR8281009
Aega_sp_KK1162_2R
Aegiochus_sp_KK1240_3C
Aegidae_IARR144
Hyloniscus_beckeri_SRR27386476_SRR27386475_SRR27386474
Trichoniscus_matulici_SRR27386479_SRR27386478_SRR27386477
Titanethes_albus_SRR27386473_SRR27386472_SRR27386471
Platyarthrus_hoffmannseggi_29120
Oniscus_asellus_SRR5298319
Philoscia_muscorum_29152
Cylisticus_convexus_29744
Trachelipus_rathkii_SRR5198726_SRR5198727
Porcellio_scaber_29156
Paraplatyarthrus_subterraneus_SRR15808559
Armadillo_officinalis_SRR5298311
Cyclaspis_cf_gigas_KK1807_3R
Vaunthompsonia_sp_KK4942_2R
Eudorella_flokkeri_KK1908_3R_KK1908_4R
Leucon_costatus_KK2234_3R_both
Campylaspis_sp_KK4059_1R
Schizocuma_sp_KK5212_1R_both
Diastylopsis_goekei_KK2103_2R
Diastylis_anderssoni_KK1805_2R
Ekleptostylis_debroyeri_KK2111_1R
Leptostylis_sp_KK4300_2C
Phoxokalliapseudes_tomiokaensis_GJCI01
Pseudosphyrapus_quintolongus_GJCK01
Pakistanapseudes_sp_GJCL01
Parapseudes_algicola_GJCR01
Carpoapseudes_spinigena_GJCJ01
Apseudes_sp_GJCG01
Paradoxapseudes_littoralis_GJCE01
Apseudomorpha_sp_GJCD01
Neotanais_cf_kuroshio_GJCN01
Arctotanais_alascensis_GJCM01
Zeuxo_ezoensis_GJCB01
Hexapleomera_sasuke_GJCH01
Chondrochelia_sp_GJCT01
Leptochelia_sp_SRR5140116
Heterotanoides_sp_GJCS01
Tanaopsis_sp_GJCV01
Exspina_typica_KK4674_4R
Agathotanais_misakiensis_GJCP01
Paranarthrura_sp_GJCW01
Parakanthophoreus_sp_GJCX01
Siphonolabrum_sp_GJCU01
Tanaella_kommritzia_GJCQ01
Akanthophoreus_sp_GJCY01
Chauliopleona_cf_sinusa_GJCO01
Mictocaris_halope_KK5680_1R_KK5681
Poticoara_brasiliensis_JO_2018_BR_23
Spelaeogriphus_sp_KK5769_1R
Allobathynella_bangokensis_SRR4198914_SRR4198915
Paranaspides_williamsi_KK5759_1R
Allanaspides_helonomus_KK5760_1R
Euphausia_superba_GFCS01
Meganyctiphanes_norvegica_GETT01
Nematoscelis_IARR489
Thysanoessa_inermis_SRR2174568
Halocaridina_rubra_SRR3089133_SRR3089132
Alpheus_cf_viridari_SRR23641092
Thor_dobkini_SRR23641091
Lysmata_wurdemanni_SRR8715486
Parasergestes_armatus_SRR13389714
Penaeus_vannamei_SRR29849178
Penaeus_monodon_GCF_015228065
Paralithodes_camtschaticus_SRR8715480
Eriocheir_sinensis_GCF_024679095
Pachygrapsus_marmoratus_SRR11077390
Panulirus_ornatus_SRR26945904
Procambarus_clarkii_GCF_020424385
Homarus_americanus_GCF_018991925
Enoplometopus_cf_daumi_SRR5140101
Pullosquilla_thomassini_SRR19721501_SRR19721502_SRR19721503_SRR19721504_SRR19721505
Neogonodactylus_oerstedii_SRR17270924
Oratosquilla_oratoria_GGQQ01
30
