# Supplementary material for: Phylogenomics supports monophyly of marsupial crustaceans: a journey to direct development
Source: Mol Biol Evol. 2026 Aug 13;43(8):msag178. doi: 10.1093/molbev/msag178 (PMC13472170; doi:10.1093/molbev/msag178)
Supplement: Supplementary file 1 [file msag178_supplementary_data.zip › S63_ORTHO50_BAYES_scf_gcf.pdf]

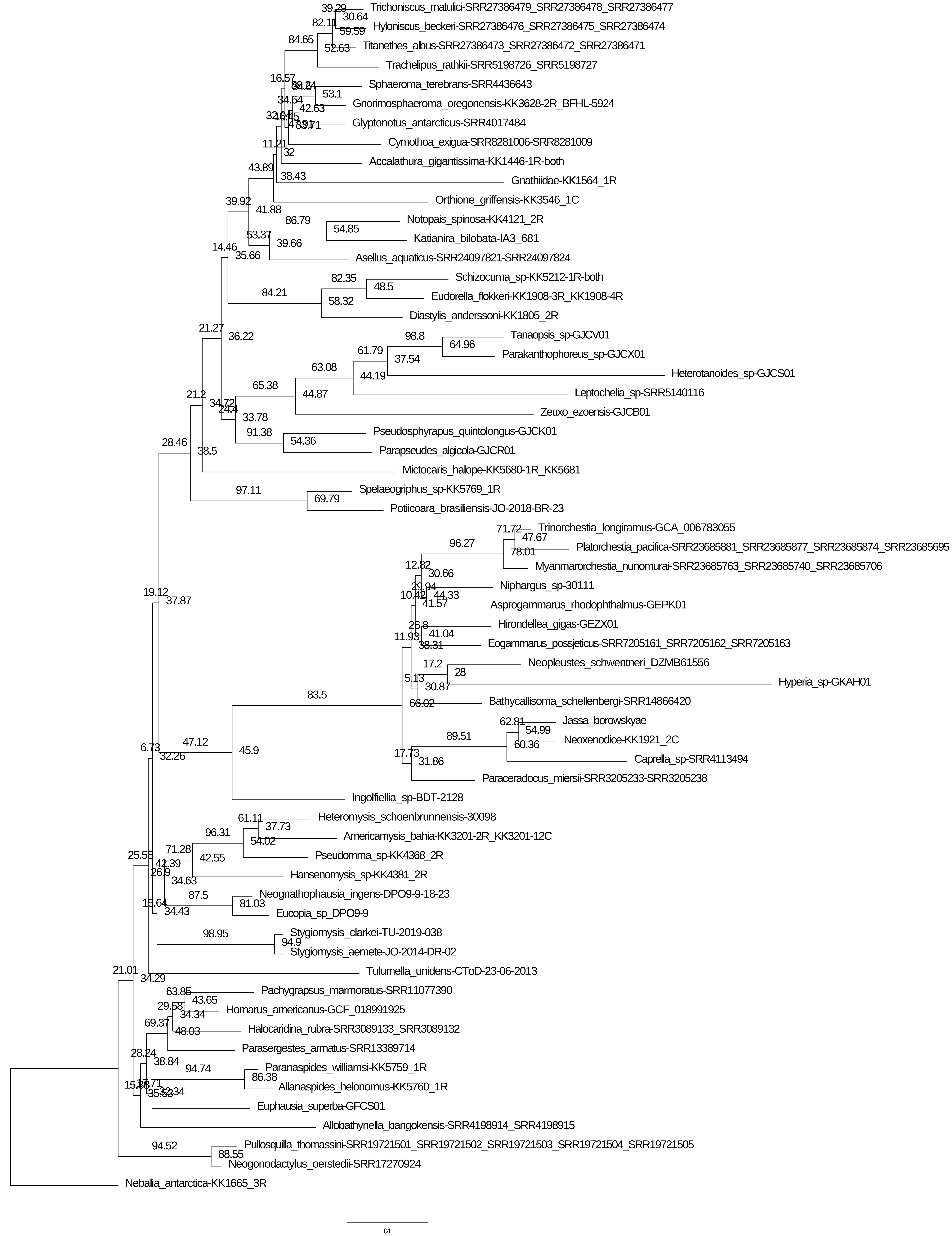
Trichoniscus_matulici-SRR27386479_SRR27386478_SRR27386477
Hyloniscus_beckeri-SRR27386476_SRR27386475_SRR27386474
Titanethes_albus-SRR27386473_SRR27386472_SRR27386471
Trachelipus_rathkii-SRR5198726_SRR5198727
Sphaeroma_terebrans-SRR4436643
Gnorimosphaeroma_oregonensis-KK3628-2R_BFHL-5924
Glyptonotus_antarcticus-SRR4017484
Cymothoa_exigua-SRR8281006-SRR8281009
Accalathura_gigantissima-KK1446-1R-both
Gnathiidae-KK1564_1R
Orthione_griffensis-KK3546_1C
Notopais_spinosa-KK4121_2R
Katianira_bilobata-IA3_681
Asellus_aquaticus-SRR24097821-SRR24097824
Schizocuma_sp-KK5212-1R-both
Eudorella_flokkeri-KK1908-3R_KK1908-4R
Diastylis_anderssoni-KK1805_2R
Tanaopsis_sp-GJCV01
Parakanthophoreus_sp-GJCX01
Heterotanoides_sp-GJCS01
Leptochelia_sp-SRR5140116
Zeuxo_ezoensis-GJCB01
Pseudosphyrapus_quintolongus-GJCK01
Parapseudes_algicola-GJCR01
Mictocaris_halope-KK5680-1R_KK5681
Spelaeogriphus_sp-KK5769_1R
Potiicoara_brasiliensis-JO-2018-BR-23
Trinorchestia_longiramus-GCA_006783055
Platorchestia_pacifica-SRR23685881_SRR23685877_SRR23685874_SRR23685695
Myanmarorchestia_nunomurai-SRR23685763_SRR23685740_SRR23685706
Niphargus_sp-30111
Asprogammarus_rhodophthalmus-GEPK01
Hirondellea_gigas-GEZX01
Eogammarus_possjeticus-SRR7205161_SRR7205162_SRR7205163
Neopleustes_schwentneri_DZMB61556
Hyperia_sp-GKAH01
Bathycallisoma_schellenbergi-SRR14866420
Jassa_borowskyae
Neoxenodice-KK1921_2C
Caprella_sp-SRR4113494
Paraceradocus_miersii-SRR3205233-SRR3205238
Ingolfiellia_sp-BDT-2128
Heteromysis_schoenbrunnensis-30098
Americamysis_bahia-KK3201-2R_KK3201-12C
Pseudomma_sp-KK4368_2R
Hansenomysis_sp-KK4381_2R
Neognathophausia_ingens-DPO9-9-18-23
Eucopia_sp_DPO9-9
Stygiomysis_clarkei-TU-2019-038
Stygiomysis_aemete-JO-2014-DR-02
Tulumella_unidens-CToD-23-06-2013
Pachygrapsus_marmoratus-SRR11077390
Homarus_americanus-GCF_018991925
Halocaridina_rubra-SRR3089133_SRR3089132
Parasergestes_armatus-SRR13389714
Paranaspides_williamsi-KK5759_1R
Allanaspides_helonomus-KK5760_1R
Euphausia_superba-GFCS01
Allobathynella_bangokensis-SRR4198914_SRR4198915
Pullosquilla_thomassini-SRR19721501_SRR19721502_SRR19721503_SRR19721504_SRR19721505
Neogonodactylus_oerstedii-SRR17270924
Nebalia_antarctica-KK1665_3R
